# *NODAL* variants are associated with a continuum of laterality defects from simple D-transposition of the great arteries to heterotaxy

**DOI:** 10.1186/s13073-024-01312-9

**Published:** 2024-04-03

**Authors:** Zain Dardas, Jawid M. Fatih, Angad Jolly, Moez Dawood, Haowei Du, Christopher M. Grochowski, Edward G. Jones, Shalini N. Jhangiani, Xander H. T. Wehrens, Pengfei Liu, Weimin Bi, Eric Boerwinkle, Jennifer E. Posey, Donna M. Muzny, Richard A. Gibbs, James R. Lupski, Zeynep Coban-Akdemir, Shaine A. Morris

**Affiliations:** 1 Department of Molecular and Human Genetics, Baylor College of Medicine, Houston, TX 77030 USA; 2 Human Genome Sequencing Center, Baylor College of Medicine, Houston, TX 77030 USA; 3 Medical Scientist Training Program, Baylor College of Medicine, Houston, TX 77030 USA; 4 Division of Cardiology, Department of Pediatrics, Texas Children’s Hospital and Baylor College of Medicine, Houston, TX 77030 USA; 5 Cardiovascular Research Institute, Baylor College of Medicine, Houston, TX 77030 USA; 6 Department of Integrative Physiology, Baylor College of Medicine, Houston, TX 77030 USA; 7 Department of Medicine, Baylor College of Medicine, Houston, TX 77030 USA; 8 Baylor Genetics, Houston, TX 77021 USA; 9 Human Genetics Center, Department of Epidemiology, Human Genetics, and Environmental Sciences, School of Public Health, The University of Texas Health Science Center at Houston, Houston, TX 77030 USA; 10 Texas Children’s Hospital, Houston, Houston, TX 77030 USA; 11 Department of Pediatrics, Baylor College of Medicine, Houston, TX 77030 USA

**Keywords:** Congenital heart disease, *NODAL*, Laterality defects, Heterotaxy, Transposition, Single ventricle, Genetic diagnosis, Structural variation

## Abstract

**Background:**

NODAL signaling plays a critical role in embryonic patterning and heart development in vertebrates. Genetic variants resulting in perturbations of the TGF-β/NODAL signaling pathway have reproducibly been shown to cause laterality defects in humans. To further explore this association and improve genetic diagnosis, the study aims to identify and characterize a broader range of *NODAL* variants in a large number of individuals with laterality defects.

**Methods:**

We re-analyzed a cohort of 321 proband-only exomes of individuals with clinically diagnosed laterality congenital heart disease (CHD) using family-based, rare variant genomic analyses. To this cohort we added 12 affected subjects with known *NODAL* variants and CHD from institutional research and clinical cohorts to investigate an allelic series. For those with candidate contributory variants, variant allele confirmation and segregation analysis were studied by Sanger sequencing in available family members. Array comparative genomic hybridization and droplet digital PCR were utilized for copy number variants (CNV) validation and characterization. We performed Human Phenotype Ontology (HPO)-based quantitative phenotypic analyses to dissect allele-specific phenotypic differences.

**Results:**

Missense, nonsense, splice site, indels, and/or structural variants of *NODAL* were identified as potential causes of heterotaxy and other laterality defects in 33 CHD cases. We describe a recurrent complex indel variant for which the nucleic acid secondary structure predictions implicate secondary structure mutagenesis as a possible mechanism for formation. We identified two CNV deletion alleles spanning *NODAL* in two unrelated CHD cases. Furthermore, 17 CHD individuals were found (16/17 with known Hispanic ancestry) to have the c.778G > A:p.G260R *NODAL* missense variant which we propose reclassification from variant of uncertain significance (VUS) to likely pathogenic. Quantitative HPO-based analyses of the observed clinical phenotype for all cases with p.G260R variation, including heterozygous, homozygous, and compound heterozygous cases, reveal clustering of individuals with biallelic variation. This finding provides evidence for a genotypic-phenotypic correlation and an allele-specific gene dosage model.

**Conclusion:**

Our data further support a role for rare deleterious variants in *NODAL* as a cause for sporadic human laterality defects, expand the repertoire of observed anatomical complexity of potential cardiovascular anomalies, and implicate an allele specific gene dosage model.

**Supplementary Information:**

The online version contains supplementary material available at 10.1186/s13073-024-01312-9.

## Background

The cardiovascular system is among the first physiological functional systems to develop in the vertebrate embryo. Heart development initiates with the formation of the primitive heart tube following the torsional folding of the embryo during the end of the third week of gestation. Once formed, the primitive heart tube must break the pre-existing left-right (L-R) symmetry and undergo a series of septation events that culminate in the formation of a four-chambered heart [1]. Subtle deviations in heart development can result in congenital heart disease (CHD), which is a collection of defects that together comprise the most prevalent form of birth defect with a birth prevalence of 0.8% of all newborns [2, 3]. The etiology of CHD is incompletely understood and certainly due to multiple mechanisms.

One large class of CHD are those related to laterality defects. Classically the laterality defect classification has included situs inversus totalis (complete mirror-image reversal of the chest and abdominal organs usual positions) and heterotaxy (a state of partial rearrangement or anatomical positioning with regard to the body axes) [4]. However, both animal and human published studies suggest that other CHD lesions could be due to altered laterality development of the heart [4–7]. In fact, 3–7% of all apparently isolated CHDs, comprising double outlet right ventricle (DORV), atrioventricular canal defect (AVCD), or transposition of the great arteries (TGA), have been suggested to arise from abnormal embryonic L-R axis patterning [5, 6].

The genetics underlying the etiology of laterality defects is heterogeneous and our understanding of the genes involved is limited, but autosomal dominant, autosomal recessive, and X-linked inheritance patterns have each been observed for rare disease traits involving heterotaxy [8, 9]. Nevertheless, most clinical cases observed are sporadic in nature and could have de novo mutation contributing or perhaps novel compound inheritance including combinations of biallelic variant alleles contributed from each parent. Over 100 genes, including *NODAL*, *ACVR2B*, *GDF1*, *ZIC3*, *SHROOM3*, *LZTFL1*, and ciliary genes like *DNAH11*, *DNAAF1*, and *ODAD1*, have been implicated in laterality defects [10].

A key player in the molecular control of L-R axis development is the NODAL signaling pathway [11]. Studies in mice revealed that during gastrulation, Nodal, a growth factor from the TGF-ß family, is asymmetrically expressed in the primitive node. Expression is expanded and amplified in the left-lateral plate mesoderm (L-LPM) but inhibited in the right-lateral plate mesoderm (R-LPM) [12].

Here, we analyzed a large cohort of individuals with clinically diagnosed laterality defects. We found evidence for missense, nonsense, splice site, indels, and/or structural variants in *NODAL* as potential causes of heterotaxy and other laterality defects in 33 cases. Furthermore, we reinvestigated ClinVar classification of *NODAL* missense variant NM_018055.5: c.778G > A:p.G260R (Conflicting Interpretation of Pathogenicity into Likely Pathogenic), which was identified in 17/33 cases, and report quantitative phenotypic comparisons of patients with G260R in heterozygous versus biallelic (homozygous and compound heterozygous) states, which implicate a gene dosage effect.

## Methods

### Case ascertainment

Cases were ascertained from 2 sources (Additional file 1: Tables S1-S3) of subjects with laterality CHD, defined as heterotaxy or congenital heart defects thought to arise by atrio-ventricular discordance or by ventriculo-arterial discordance. Group 1 is a large prospective study of laterality CHD (*n* = 583) for which probands were recruited with informed consent to undergo genetic testing based at Baylor College of Medicine (IRB approval number: H-1843). Within this study, 321 of the subjects underwent proband-only exome sequencing (ES) by the Center for Mendelian Genomics (Group 1a). Second (Group 1b), available relevant family members of those found to have a *NODAL* variant in Group 1a underwent targeted testing. Third, a smaller number of subjects (*n* = 269, Group 1c) within Group 1 with some but not complete overlap of Group 1a had previously undergone single-gene sequencing for *NODAL* variants [13]. Group 2 is comprised of probands and family members from Texas Children’s Hospital Heart Center with laterality CHD in which the proband underwent microarray or exome or panel sequencing (including *NODAL*) which demonstrated pathogenic/likely pathogenic (P/LP) *NODAL* variants and had CHD were included. For one proband in Group 2, a CNV spanning *NODAL* was discovered upon reanalysis using the clinical microarray (CMA) data available at Baylor Genetics (BG) (CVG0007). The Institutional Review Board of Baylor College of Medicine approved all research study protocols. Written informed consent was obtained from all participating individuals including probands and any available family members from Group 1. For the clinically tested patients (Group 2), a waiver of consent was granted as part of 2 retrospective cohort studies of clinical genetic testing in CHD (IRB approval numbers: H-48014 and H-41191). Clinical data were ascertained by individual and familial history, as well as review of the medical records. Cardiac phenotypic data were obtained by the review of echocardiograms, magnetic resonance imaging, computed tomography imaging, angiography, and operative reports.

### Exome sequencing

ES was performed on genomic DNA for probands from Group 1a at the Human Genome Sequencing Center at Baylor College of Medicine through the Baylor-Hopkins Center for Mendelian Genomics initiative using the Illumina HiSeq 2000 platform and the Mercury pipeline as described previously [14, 15]. The methods used for Group 1c (*n* = 269) are described in Mohapatra et al. [13]. For those *NODAL* variant subjects with CHD ascertained through clinical sequencing, all 5 underwent CMA. Regarding *NODAL* sequencing, two of the clinical cases underwent trio exome sequencing (CVG0005-fetal and CVG0006), one underwent proband-only sequencing due to unavailability of parental samples (CVG0001), one underwent a trio laterality panel (CVG0003), and one underwent a proband-only laterality panel (CVG0007).

### Variant filtering and validation

To detect potential disease-causing *NODAL* SNVs and indels in exome and panel sequencing, a stepwise analysis workflow was implemented. We investigated homozygous, heterozygous, and compound-heterozygous variant alleles from a 329 gene list (Additional file 1: Table S4) that includes either known or candidate CHD genes. These genes were collated by combining the genes in the CHD gene database [16], genes listed on the cardiology panels of Baylor Genetics, Invitae, and Ambry, as well as including known human CHD genes and CHD genes involved in mouse experiments. Rare variants (< 0.01%) were prioritized according to frequency in the population databases including the 1000 Genomes Project (TGP); the Atherosclerosis Risk in Communities Study Database (ARIC); gnomAD; and our in-house-generated exome database (personal genome exomes from ∼13,000 individuals) at the BCM-HGSC. Rare variants with a Combined Annotation Dependent Depletion (CADD)-phred score of > 15 were included. Candidate SNVs that remained after the ES analysis process were orthogonally validated and segregated in available family members via an orthogonal approach (Sanger- Dideoxy sequencing).

### DNA cloning

To validate the indel variant (p.R234_P241delinsLTS) in families (6 and 7), we performed cloning experiments using the TA Cloning™ Kit (Catalog number: K202020) from Invitrogen™. Primers were designed to amplify a target region (525 bp) flanking the variant. A ligation reaction between pCR2.1 and the target gene was set up with a 1:1 vector/insert molar ratio. The ligation reaction was carried out in 5X T4 DNA ligase reaction buffer at 25 °C for 1 h. Two microliters of the ligation reaction was used to transform 50 μl of One Shot™ TOP10 Chemically Competent *E. coli* cells (Catalog number: C404003). The transformed cells were single colony purified on Luria-Bertani (LB) plates containing kanamycin and incubated at 37 °C for 16 h. Twelve colonies were randomly selected, inoculated into LB broth, and cultured overnight. Sanger dideoxy sequencing was performed on the cultured colonies.

### *NODAL* CNV analysis

To identify potential CNV deletions spanning *NODAL* from exome data, we used XHMM (eXome-Hidden Markov Model) [17], a publicly available bioinformatics tool, and an in-house-developed software, HMZDelFinder [18]. Furthermore, CNVs spanning *NODAL* were assessed using the CMA data available at BG.

### Human Phenotype Ontology (HPO)-based quantitative phenotypic similarity analysis

A detailed description of the methods used for HPO-based quantitative phenotypic similarity analysis has been previously published [19]. Briefly, proband cardiac and laterality phenotypes were annotated using HPO terms (Additional file 1: Table S5). A symmetric Lin similarity score was calculated with the OntologyX suite of R packages [20] and used to generate pairwise phenotypic similarity scores between all *NODAL* probands. This similarity matrix was then used to generate a distance matrix for clustering analysis. A gap statistic was calculated for number of clusters 1–15 and plotted to generate a gap statistic curve. The slope of the curve, namely the point at which the slope of the curve had the greatest decrease (4 clusters) was used as a guide for the number of clusters to group probands into. Hierarchical Agglomerative Clustering (HAC) using the Ward method was then used to cluster probands. A heatmap was generated with the ComplexHeatmap [21] R package based on the previously generated similarity matrix and ordered according to HAC clustering.

### Orthogonal validation of predicted *NODAL* CNV deletions

Droplet digital PCR (ddPCR) and/or array comparative genomic hybridization (aCGH) were performed for variant copy number validation and segregation analysis of two potential *NODAL* CNV deletions in two unrelated families. ddPCR experiments were performed using the QX200 AutoDG Droplet Digital PCR System according to the manufacturer’s protocols and previously described methods [22] (Additional file 2: Table S6 for primer information).

For aCGH, we used an Agilent custom-designed high-resolution array targeting Chr10q (AMADID: 086730). Microarray protocols, including DNA digestion, probe labeling, gender-matched hybridization, and post-washing, were performed as described previously with minor modifications [23]. Agilent SureScan and Feature Extraction software were utilized to achieve the image-to-digital transition, with further data analysis and visualization on the Agilent Genomic Workbench. Genomic coordinates were described in reference to GRCh37/hg19 assembly.

### Breakpoint junction analysis of *NODAL* CNV deletions

Breakpoint junctions identified in the aCGH data were located and visualized using the Agilent Genomic Workbench. Inward facing primers were designed outside of the deleted regions. Breakpoint junctions were obtained through long-range PCR (LR-PCR) using TaKaRa LA Taq according to the manufacturer’s protocol (TaKaRa Bio Company, Cat.No.RR002). Purified PCR products were sequenced by Sanger dideoxy sequencing (BCM Sequencing Core, Houston, TX, USA). To map the nucleotide-level positions of the breakpoint junctions, the DNA sequences resulting from Sanger sequencing were aligned to the reference genome sequence (UCSC genome browser, GRCh37/hg19).

## Results

### Genotypic and phenotypic expansion of *NODAL* SNVs/INDEL in laterality defects

The majority of cases were collected from a prospective study of congenital cardiac laterality defects. Proband exome sequencing was available in 321 of these subjects. While a *NODAL* SNV or indel had been already reported in 10 of these subjects by our group [13, 15], re-analysis detected an additional 11 cases, for a total of 21/321 (6.5%) cases with a *NODAL* variant and laterality CHD. An additional 6 cases with a *NODAL* variant were included that were previously reported by our group using single-gene sequencing for *NODAL* of probands from a separate subcohort of laterality CHD [13]. For these 27 probands from Group 1 and the 4 probands from Group 2 who had incomplete familial testing, we performed Sanger dideoxy sequencing for variant allele confirmation and segregation analysis in available family members. The *NODAL* variant was inherited in 19 (including 3 homozygous and 1 compound heterozygous inheritance), de novo in 2, and inheritance data unknown in 10 probands because of lacking information from one or both parents.

**Fig. 1:**
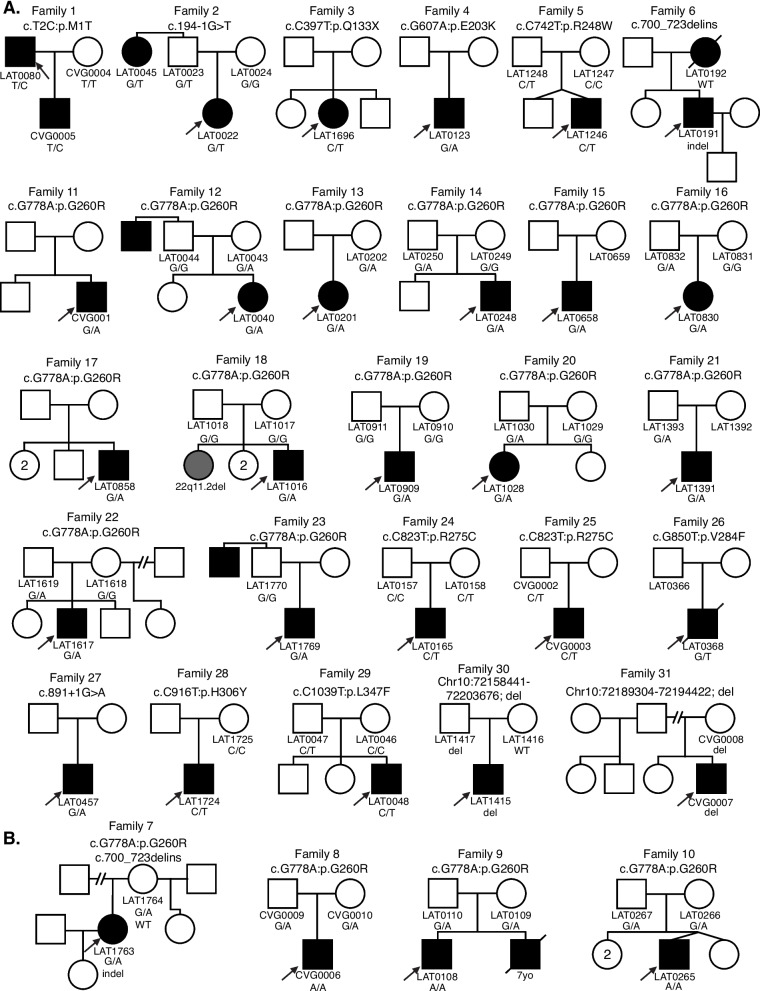
Comprehensive pedigrees and their genotypes for families with *NODAL* variants. Standard pedigree structures are utilized—filled circles and squares denote clinically affected individuals, and probands are indicated by black arrows. **A** Pedigrees of probands harboring heterozygous *NODAL* variants. **B** Pedigrees of probands harboring biallelic *NODAL* variants

Analysis of relatives also revealed an unreported case of an affected aunt (LAT0045, Family 2) with laterality CHD and a *NODAL* variant (Fig. 1). In Group 2, one infant subject that underwent clinical testing (CVG0005) was a son of a proband (LAT0080) in the larger 321-person (Group 1a). These combined cohorts and parental evaluation resulted in 31 unrelated families with *NODAL* variants; 33 subjects with CHD (Additional file 1: Tables S1 and S2).

**Fig. 2:**
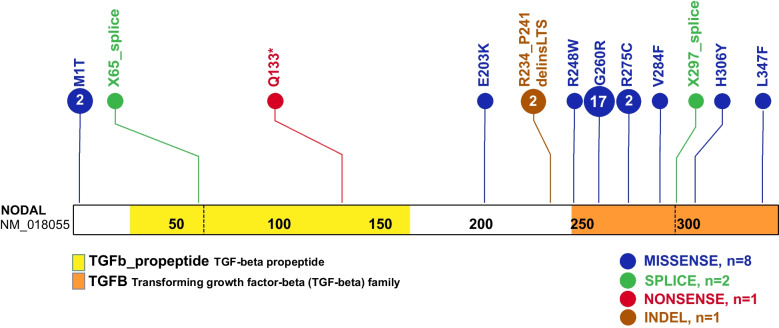
Schematic diagram of NODAL protein structure from conceptual translation of transcript NM_018055 with mapping location (vertical lollipops) of the exact map position of amino acid variants observed in this study. In total, we identified twelve different SNV alleles and indel that are distributed among the NODAL domains. The yellow horizontal rectangle represents the TGF-beta propeptide (amino acid 29-166) and the orange horizontal rectangle represents the mature transforming growth TGF-beta domain (amino acid 247-347). Blue circles denote missense variants, green circles denote splice site variants, red circles denote nonsense variants, and brown circles denote a complex indel variant. Numbers inside the circles refer to the variant frequency in our cohort

A total of eleven *NODAL* SNVs (8 missense, 2 splice site, and 1 nonsense) were detected in 31 laterality CHD cases from 29 families (Table 1, Fig. 1). These variant alleles mainly were localized to the portions of the protein constituting the TGF-β mature domain (Fig. 2). Cases with *NODAL* variants showed various CHD lesions, all of which had abnormal ventricular looping and/or abnormal great artery relationship (Table 2—summary, Additional file 1: Table S3-detailed). Within the laterality cohort, *NODAL* variants were most frequently observed in cases with congenitally corrected transposition of the great arteries (CCTGA), also known as levo-transposition of the great arteries (LTGA) (15.4%) and least frequently in cases with simple dextro-transposition of the great arteries (DTGA) (6.1%) and double outlet right ventricle with malposition of the great arteries (0.0%) (Table 3).

**Table 1:** *NODAL* variation identified in congenital cardiac laterality defects

**(a) Single-nucleotide variations and INDELs**																
**ID**	**Family #**	**Report**	**Sex**	**Ethn.**	**Inh.**	**Zyg.**	**Genomic position**	**C change**	**P change**	**Mut.**	**vR**	**tR**	**gnomAD**	**CADD_phred**	**REVEL**	**ClinVar**
CVG005	Family 1	This study	M	NHW	Pat.	Het	10:72201422; A > G	c.2T>C	p.M1T	ms	5	12	.	24.9	0.62	NF
LAT0080		This study	M	NHW	Unk	Het	10:72201422; A > G	c.2T>C	p.M1T	ms	5	12	.	24.9	0.62	NF
LAT0022	Family 2	Li&Moh	F	NHW	Pat.	Het	10:72195740; C > A	c.194-1G > T	-	sp	36	65	.	24		LP
LAT0045		Li&Moh	F	NHW	Unk	Het	10:72195740; C > A	c.194-1G > T	-	sp				24		LP
LAT1696	Family 3	Li	F	NHW	Unk	Het	10:72195536; G > A	c.397C>T	p.Q133^*^	ns	40	81	4.06E-06	28.4	-	P
LAT0123	Family 4	Moh	M	NHA	Unk	Het	10:72195326; C > T	c.607G>A	p.E203K	ms	43	86	0.0004	11.7	0.45	B/LB
LAT1246	Family 5	This study	M	NHW	Pat.	Het	10:72195191; G > A	c.742C>T	p.R248W	ms	87	188	.	32	0.69	US
LAT0191	Family 6	Li&Moh	M	H	Unk	Het	10:72195210–72195233; delins	c.700_723delinsT TGACTTCC	p.R234_P241 delinsLTS	indel	.	.	.	.	.	US
**LAT1763**	**Family 7**	**Li**	**F**	**H**	**Unk**	**Het**	**10:72195210–72195233; delins**	**c.700_723delinsT** **TGACTTCC**	**p.R234_P241** **delinsLTS**	**indel**	**.**	**.**	**.**	**.**	**.**	**US**
					**Mat.**	**Het**	**10:72195155; C > T**	**c.778 G> A**	**p.G260R**	**ms**	**68**	**126**	**0.0003**	**33**	**0.79**	**US/LP**
**CVG0006**	**Family 8**	**This study**	**M**	**H**	**AR**	**Hom**	**10:72195155; C > T**	**c.778G>A**	**p.G260R**	**ms**			**0.0003**	**33**	**0.79**	**US/LP**
**LAT0108**	**Family 9**	**This study**	**M**	**H**	**AR**	**Hom**	**10:72195155; C > T**	**c.778G>A**	**p.G260R**	**ms**	**142**	**143**	**0.0003**	**33**	**0.79**	**US/LP**
**LAT0265**	**Family 10**	**This study**	**M**	**H**	**AR**	**Hom**	**10:72195155; C > T**	**c.778G>A**	**p.G260R**	**ms**	**148**	**149**	**0.0003**	**33**	**0.79**	**US/LP**
CVG0001	Family 11	This study	M	H	Unk	Het	10:72195155; C > T	c.778G>A	p.G260R	ms			0.0003	33	0.79	US/LP
LAT0040	Family 12	Moh	F	H	Mat.	Het	10:72195155; C > T	c.778G>A	p.G260R	ms			0.0003	33	0.79	US/LP
LAT0201	Family 13	Moh	F	H	Mat.	Het	10:72195155; C > T	c.778G>A	p.G260R	ms	91	185	0.0003	33	0.79	US/LP
LAT0248	Family 14	Moh	M	H	Pat.	Het	10:72195155; C > T	c.778G>A	p.G260R	ms	49	115	0.0003	33	0.79	US/LP
LAT0658	Family 15	Moh	M	H	Unk	Het	10:72195155; C > T	c.778G>A	p.G260R	ms			0.0003	33	0.79	US/LP
LAT0830	Family 16	This study	F	H	Pat.	Het	10:72195155; C > T	c.778G>A	p.G260R	ms	56	147	0.0003	33	0.79	US/LP
LAT0858	Family 17	Moh	M	H	Unk	Het	10:72195155; C > T	c.778G>A	p.G260R	ms			0.0003	33	0.79	US/LP
LAT1016	Family 18	Moh	M	H	de novo	Het	10:72195155; C > T	c.778G>A	p.G260R	ms			0.0003	33	0.79	US/LP
LAT0909	Family 19	Moh	M	NHW	de novo	Het	10:72195155; C > T	c.778G>A	p.G260R	ms			0.0003	33	0.79	US/LP
LAT1028	Family 20	Moh	M	H	Pat.	Het	10:72195155; C > T	c.778G>A	p.G260R	ms	91	173	0.0003	33	0.79	US/LP
LAT1391	Family 21	This study	M	H	Pat.	Het	10:72195155; C > T	c.778G>A	p.G260R	ms	86	184	0.0003	33	0.79	US/LP
LAT1617	Family 22	This study	M	H	Pat.	Het	10:72195155; C > T	c.778G>A	p.G260R	ms	54	121	0.0003	33	0.79	US/LP
LAT1769	Family 23	This study	M	H	Unk	Het	10:72195155; C > T	c.778G>A	p.G260R	ms	53	108	0.0003	33	0.79	US/LP
LAT0165	Family 24	Moh	M	H	Mat.	Het	10:72195110; G > A	c.823C>T	p.R275C	ms	50	107	4.47E-05	34	0.91	P
CVG0003	Family 25	This study	M	NHW	Pat.	Het	10:72195110; G > A	c.823C>T	p.R275C	ms	50	107	4.47E-05	34	0.91	P
LAT0368	Family 26	Moh	M	NHW	Unk	Het	10:72195083; C > A	c.850G>T	p.V284F	ms			0.00003	24	0.48	NF
LAT0457	Family 27	Moh	M	H	Unk	Het	10:72195041; C > T	c.891 + 1G > A	-	sp			.	33	-	LP
LAT1724	Family 28	This study	M	NHB	Unk	Het	10:72192820; G > A	c.916C>T	p.H306Y	ms	55	100	0.0001	23.3	0.23	LB
LAT0048	Family 29	This study	M	NHW	Pat.	Het	10:72192697; G > A	c.1039C>T	p.L347F	ms	106	208	4.06E-06	31	0.51	NF
**(b) Copy number variants (CNV)**																
**ID**		**Report**	**Sex**	**Ethn.**	**Inh.**	**Zyg.**	**Genomic position**	**Size (bp)**	**Genes within CNV**	***NODAL* exons deletion**	**SV_freq**^**a**^	**SV_mut_mech**	**ClinVar**			
LAT1415	Family 30	This study	M	NHA	Pat.	Het	10:72204016–72157996; del	46020	*EIF4EBP2 & NODAL*	1–3	0	AAMR	NF			
CVG0007	Family 31	This study	M	NHW	Mat.	Het	10:72189745–72194422; del	4221	*NODAL*	3	0	AAMR	NF			

Li et al. (2019) [15] Eur. J. Hum. Genet., Mohapatra et al. (2009) [13] Hum. Mol. Genet., *AAMR Alu-Alu* Mediated Rearrangement, *B* Benign, *Ethn.* Ethnicity, *F* Female, *fs* Frameshift, *H* Hispanic, *Het* Heterozygous, *Hom* Homozygous, *indel* Insertion-deletion, *Inh.* Inheritance, *LB* Likely benign, *LP* Likely pathogenic, *NF* Not found, *NHA* Non-Hispanic Asian, *NHB* Non-Hispanic black, *NHW* Non-Hispanic white, *ns* Nonsense, *Mat* Maternal, *M* Male, *Mut*. Mutation type, *ms* Missense, *P* Pathogenic, *Pat* Paternal, *sp* Splicing, *tR* Total read, *Unk* Unknown, *US* Unknown significance, *vR* Variant read, *Zyg.* Zygosity

^a^SV frequencies are reported as the frequency of deletions in gnomAD that completely overlap the deletion CNV found in the proband. Probands with biallelic variants are highlighted in bold

**Table 2:** Summarized clinical information

**Sample ID**	**Family #**	**Report**^**a**^	**Sex**	**Race/Ethnicity**	**Zygosity**	**C change**	***P* change**	**Seg. anatomy**^b^	D-looped ventricles			L-looped ventricles			Systemic laterality condition	
									**Simple DTGA**	**DORV with D-MGA**	**Tricuspid atresia with MGA**	**CCTGA**	**DILV**	**Other left ventricular looping lesion**	**Heterotaxy (right atrial isomerism/asplenia)**	**Situs inversus**
CVG005	Family 1	This study	M	NHW	Het	c.2T > C	p.M1T	S,L,L				X				
LAT0080		This study	M	NHW	Het	c.2T > C	p.M1T	A,D,D							X	
LAT0022	Family 2	Li&Moh	F	NHW	Het	c.194-1G > T	N/A	S,L,L					X			
LAT0045		This study	F	NHW	Het	c.194-1G > T	N/A					X				
LAT1696	Family 3	Li	F	NHW	Het	c.397C > T	p.E133K	S,L,L				X				
LAT0123	Family 4	Moh	M	NHA	Het	c.607G > A	p.E203K	I,L,D								X
LAT1246	Family 5	This study	M	NHW	Het	c.742C > T	p.R248W	S,L,L				X				
LAT0191	Family 6	Li&Moh	M	H	Het	c.700_723delins	p.R234_P241 delinsLTS	S,L,L					X			
**LAT1763**	**Family 7**	**Li**	**F**	**H**	**Het**	**c.700_723delins**	**p.R234_P241 delinsLTS**	**A,D,D**							**X**	
		**This study**			**Het**	**c.778G.A**	**p.G260R**									
**CVG006**	**Family 8**	**This study**	**M**	**H**	**Hom**	**c.778G > A**	**p.G260R**	**A,D,D**							**X**	
**LAT0108**	**Family 9**	**This study**	**M**	**H**	**Hom**	**c.778G > A**	**p.G260R**	**A,D,D**							**X**	
**LAT0265**	**Family 10**	**This study**	**M**	**H**	**Hom**	**c.778G > A**	**p.G260R**	**A,?,D**							**X**	
CVG001	Family 11	This study	M	H	Het	c.778G > A	p.G260R	S,L,L						X		
LAT0040	Family 12	Moh	F	H	Het	c.778G.A	p.G260R	I,D,D								X
LAT0201	Family 13	Moh	F	H	Het	c.778G > A	p.G260R	S,L,X					X			
LAT0248	Family 14	Moh	M	H	Het	c.778G > A	p.G260R	A,D,D							X	
LAT0658	Family 15	Moh	M	H	Het	c.778G > A	p.G260R	A,?,X							X	
LAT0830	Family 16	This study	F	H	Het	c.778G > A	p.G260R	S,L,D						X		
LAT0858	Family 17	Moh	M	H	Het	c.778G > A	p.G260R	S,D,D		X						
LAT1016	Family 18	Moh	M	H	Het	c.778G > A	p.G260R	S,D,D		X						
LAT0909	Family 19	Moh	M	NHW	Het	c.778G > A	p.G260R	S,D,D	X							
LAT1028	Family 20	Moh	F	H	Het	c.778G > A	p.G260R	S,D,L					X			
LAT1391	Family 21	This study	M	H	Het	c.778G > A	p.G260R	S,D,D	X							
LAT1617	Family 22	This study	M	H	Het	c.778G > A	p.G260R	S,L,L				X				
LAT1769	Family 23	This study	M	H	Het	c.778G > A	p.G260R	S,L,L				X				
LAT0165	Family 24	Moh	M	H	Het	c.823C > T	p.R275C	A,D,D							X	
CVG003	Family 25	This study	M	NHW	Het	c.823C > T	p.R275C	S,D,L			X					
LAT0368	Family 26	Moh	M	NHW	Het	c.850G > T	p.V284F	S,L,L				X				
LAT0457	Family 27	Moh	M	H	Het	c.891 + 1 G > A	N/A	S,L,L						X		
LAT1724	Family 28	This study	M	NHB	Het	c.916C > T	p.H306Y	A,D,X							X	
LAT0048	Family 29	This study	M	NHW	Het	c.1039C > T	p.L347F	S,D,D	X							
LAT1415	Family 30	This study	M	NHA	Het	CNV	CNV: del 10:72204016–72157996	S,D,D			X					
CVG007	Family 31	This study	M	NHW	Het	CNV	CNV: del 10:72189745– 72194422	A,L,L							X	

The rows in bold represent biallelic cases

*CCTGA* Congenitally corrected transposition of the great arteries, *CNV* Copy number variant, *DILV* Double inlet left ventricle, *D-MGA* Dextro-malposed great arteries, *DORV* Double outlet right ventricle, *DTGA* Dextro-transposition of the great arteries, *F* Female, *H* Hispanic, *Het*. Heterozygous, *Hom*. Homozygous, *M* Male, *NHA* Non-Hispanic Asian, *NHW* Non-Hispanic White

^a^Li et al. (2019) [15] Eur. J. Hum. Genet., Mohapatra et al. (2009) [13] Hum. Mol. Genet

^b^Segmental anatomy using Van Praagh classification system [24]

**Table 3:** Frequency calculation of patients with a *NODAL* variant by CHD lesion within laterality cohort (321 probands, Group 1a)

**Lesion**	**Total**	**Total w/ *NODAL* variant**	**% with *NODAL* variant**
Simple DTGA	49	3	6.1%
DORV with malposed GA	26	0 (2 in Mohapatra paper[13]	0.0%
CCTGA	26	4	15.4%
DILV, All	33	4	12.2%
DILV, D-looped	11	1	9.1%
DILV, L-looped	22	3	13.6%
Any L-looping	66	9	13.6%
Heterotaxy—Situs inversus with CHD	15	1	6.7%
Heterotaxy—Right atrial isomerism/Asplenia syndrome	68	7	10.3%

Table only reports cases within Group 1a, which was a consecutively recruited cohort of subjects with laterality congenital heart disease

*CCTGA* Congenitally corrected transposition of the great arteries, *CHD* Congenital heart disease, *DILV* Double inlet left ventricle, *DORV* Double outlet right ventricle, *DTGA* D-Transposition of the great arteries

**Fig. 3:**
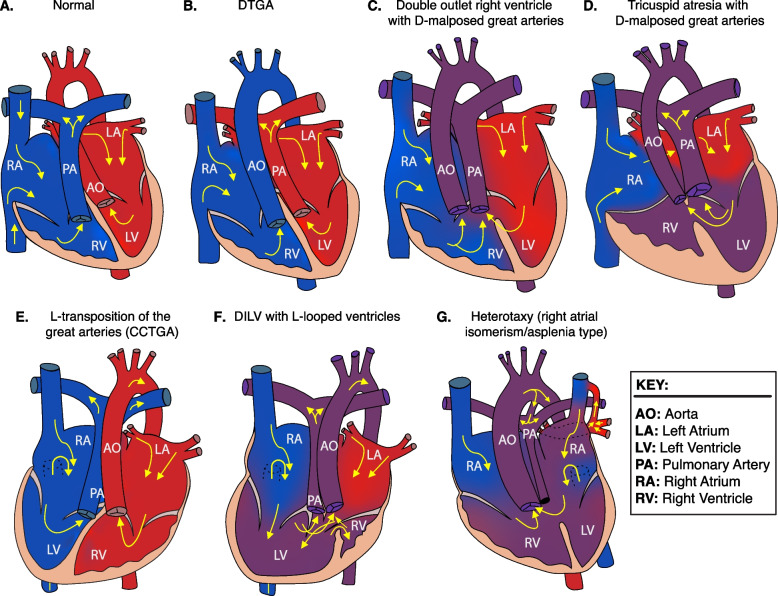
Heart depictions illustrating anatomy, blood flow (yellow arrows) and oxygenation (color coded red for oxygenated, blue for unoxygenated, and purple for poorly oxygenated) for different heart defects compared to **A** the normal heart anatomy. These include **B** dextro-transposition of the great arteries (DTGA), **C** double outlet right ventricle with D-malposed great arteries, **D** tricuspid atresia with D-malposed great arteries, **E** congenitally corrected transposition of the great arteries (CCTGA) with left ventricular looping and L-transposition of the great arteries, **F** double inlet left ventricle (DILV) with L-looped ventricles. CCTGA and DILV are illustrated with dextrocardia, but may be levocardic or dextrocardic. **G** Heterotaxy, asplenia syndrome/right atrial isomerism type

Two of the identified *NODAL* missense variants (c.2T>C, p.M1T; c.1039C>T, p.L347F) represented unreported variant alleles that have not been previously associated with laterality defects. The heterozygous p.M1T, identified in proband (LAT0080), was transmitted to his affected son (CVG005). This variant was associated with intrafamilial variable phenotypic expressivity in the proband and his son. The proband LAT0080 presented with heterotaxy, asplenia, right atrial isomerism, mitral atresia, ventricular septal defect (VSD), DORV with D-malposed great arteries (D-MGA), and pulmonary stenosis (PS). He also developed severe arteriovenous malformations (AVMs) and vesicoureteral reflux (VUR); the latter for which he underwent post ureteral reimplantation surgery. Whereas his son (CVG005) exhibited CCTGA with L-ventricular looping, LTGA, pulmonary atresia, and VSD (Table 2 and Fig. 3).

The case LAT0048, harboring a previously unreported p.L347F variant, presented with DTGA. Parental testing revealed that this rare variant was inherited from his father without CHD (Fig. 1). Leucine 347 is the last amino acid in the NODAL protein, and its substitution to phenylalanine is predicted to have a deleterious effect on NODAL function with a CADD score of 31 and a REVEL score of 0.51.

### A recurrent indel allele potentially caused by secondary structure mutagenesis

**Fig. 4:**
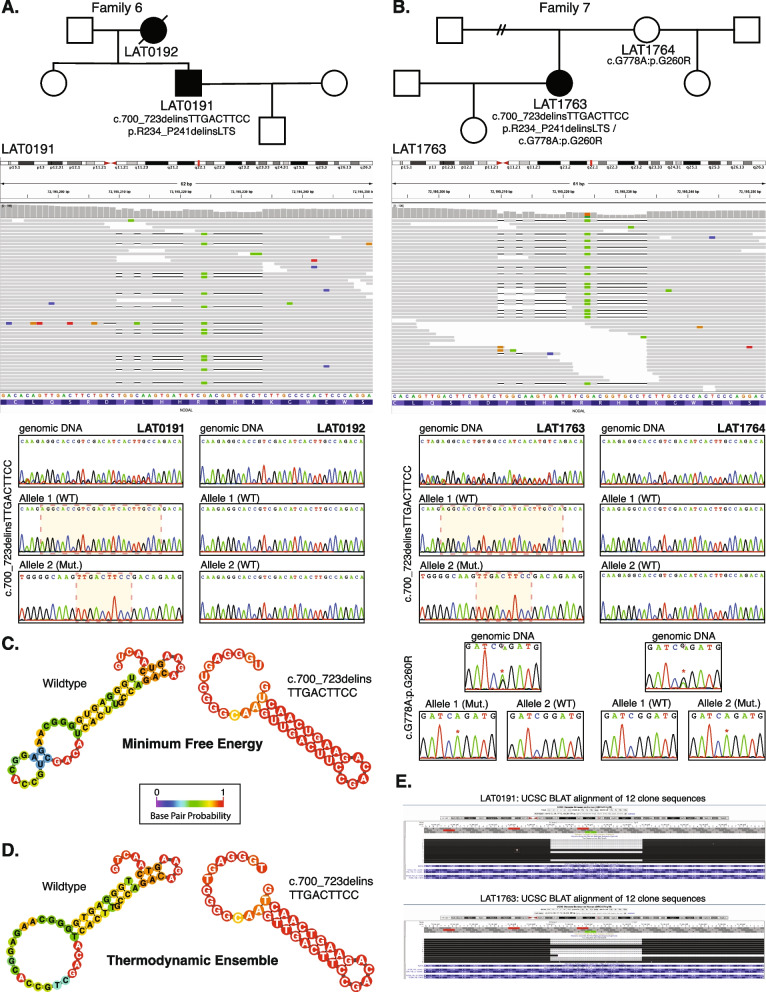
Complex *NODAL* indel variants in two probands with congenital heart disease. **A** Pedigree structure for Family 6 (top) with integrative genomics viewer (IGV) view of the heterozygous complex indel variant in *NODAL* (below). Under the IGV view are Sanger dideoxy sequence traces for the proband, LAT0191 (left) and mother, LAT0192, (right). The Sanger sequence trace panels at top represent PCR amplification products of the variant region from genomic DNA. Individual alleles are not discernable for the proband genomic DNA trace at left. Beneath the genomic DNA results are Sanger sequence traces from cloning the amplified variant region using TA cloning kits as described in “Methods”. Top and bottom panels are representative of different populations of clones for each allele. At left, the complex c.700_723delinsTTGACTTCC, p.R234_P241delinsLTS variant is apparent in the Allele 2 (Mut) Sanger trace for the proband, LAT0191. **B** Pedigree, IGV view, and Sanger traces for Family 7. In addition to the c.700_723delins, p.R234_P241delinsLTS variant, Sanger traces for the nearby c.778G > A:p.G260R variant are shown. Allele 1 in LAT1763 is shown to be WT for the c.700_723 locus, while containing the c.778G > A variant. Allele 2 was shown to contain the c.700_723delins, p.R234_P241delinsLTS variant while being WT at the c.778G > A locus, confirming these variant alleles are in a *trans* configuration. **C**, **D** Secondary structure predictions for WT and mutant RNA using the RNAfold Server (http://rna.tbi.univie.ac.at/) using the minimum free energy (**C**) and thermodynamic ensemble (**D**) functions. Base pair probability is shown using color coding; cooler colors (blue) represent lower probability and warmer colors (red) represent higher probability. Variant RNA has more stable secondary structure as shown at right in **C** and **D**. **E** UCSC genome browser view of the 12 clones sequenced for LAT0191 and LAT1763, showing populations with the deletion, and with WT sequence. All variant data shown is for *NODAL* transcript NM_018055.5

We identified one indel variant (p.R234_P241delinsLTS, c.700_723delins) in two unrelated cases (LAT0191 and LAT1763; families 6 and 7, respectively) (Table 1 and Fig. 4A, B). To facilitate variant interpretation and validation, we performed cloning experiments, to enable separation of alleles, on the probands and parental DNA for subsequent Sanger sequencing. These experiments confirmed that these two probands did not have frameshift variants, but indeed have the nonframeshift delinsLTS variant allele. Of note, the secondary structure predictions for “wild-type” (WT) and mutant single-strand nucleic acid using the RNAfold Server available at http://rna.tbi.univie.ac.at/, applying the minimum free energy (Fig. 4C) and thermodynamic ensemble (Fig. 4D) functions for intramolecular W-C base pairing, suggest that this complex mutation is mediated by secondary structure mutagenesis. The WT structure has a minimum free energy of −10.40 kcal/mol, while the mutant structure has a minimum free energy of −13.20 kcal/mol (lower by 2.80 kcal/mol). For Family 7 (LAT1763), in addition to the c.700_723delins variant allele, we also detected the c.778G > A:p.G260R variant. Allele 1 in LAT1763 is shown to be WT for the c.700_723 locus, while containing the c.778G > A variant. Allele 2 was shown to contain the c.700_723delins variant while being WT at the c.778G > A locus, confirming these variant alleles are in a *trans* configuration and thus represent a compound heterozygous combination of biallelic variation (Fig. 4).

### The G260R variant observed primarily in Hispanic ancestry heterotaxy subjects

The most common variant in the study was the c.778G > A, G260R variant, detected in 17/31 CHD probands, of which all but 1 were of known Hispanic ancestry (Additional file 2: Table S7). To compare frequencies of this G260R variant allele to that observed in the “normotypical” population (https://gnomad.broadinstitute.org/variant/10-72195155-C-T?dataset=gnomad_r2_1), we focused on the largest cohort of 321 unrelated probands with laterality CHD that were recruited consecutively (Additional file 2: Table S8). Of these, 111 self-identified as fully or partially Hispanic (35%). The *NODAL* c.778G > A, G260R variant is by far most common in Hispanic patients, with an allele frequency of 0.00219 (76/34,592 exomes in gnomAD), with no homozygotes observed. Given this frequency, we would expect ≤ 1 G260R variant in the subcohort of 111 Hispanic laterality probands. However, we identified 10 Hispanic cases within this subcohort with a *NODAL* G260R variant allele (10/111 = 0.090), and two of these cases were biallelic for the G260R variant. That gives an odds ratio of 26.0 (95%CI 13.9–48.4, *p* < 0.0001) for the cohort. Moreover, if limiting the Hispanic subcohort to the most commonly reported *NODAL*-associated laterality defects (DTGA, CCTGA, DILV, other L-ventricular looping, and heterotaxy right atrial isomerism type in the cohort), 10 had the G260R variant (10/64 = 15.6%, 12/128 alleles). So, in that group of defects, the odds ratio comparing to the gnomAD population for the G260R variant is 51.4 (95%CI 27.8–95.2, *p* < 0.0001).

### Penetrance and phenotypic variability observed with the heterozygous and homozygous G260R allele

**Fig. 5:**
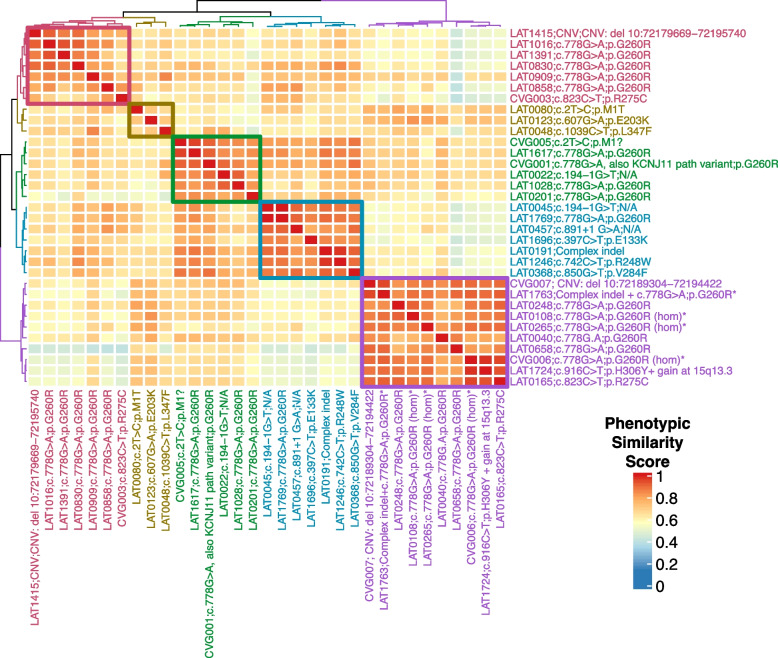
*NODAL* proband phenotypic similarity heatmap—A heatmap was generated using proband phenotypic similarity scores and ordered based on Hierarchical Agglomerative Clustering of proband phenotypic similarity. Dendrograms showing clusters are present at the left and top sides of the heatmap. Proband IDs and variants are shown at right and bottom, and color coded based on cluster. Five clusters are highlighted by color-coded boxes on the heatmap, from top left diagonally to bottom right: red, olive, green, teal, and purple. The 4 probands with biallelic variation group in one cluster (purple box). A key for phenotypic similarity score based on color (blue corresponding to a lower score and red corresponding to a higher score) is shown at bottom right. An asterisk denotes probands with biallelic variants in *NODAL*

To better understand the phenotypic spectrum associated with potentially pathogenic *NODAL* alleles, we performed quantitative phenotypic analysis of all *NODAL* cases with CHD using HPO terms. However atrial septal defect, ventricular septal defect, single ventricle, and secundum atrial septal defect were excluded from the primary analysis due to the nonspecificity of these terms to rare CHD. A supplementary analysis with these terms included is provided for comparison (Additional file 3: Fig.S1). A gap statistic curve (Additional file 3: Fig.S2) was used to determine the number of groups to cluster the cases into, and a heatmap of phenotypic similarity scores and clustering was generated using HAC (Fig. 5).

The phenotypic similarity scores among all clusters were consistently high. Notably, probands with homozygous G260R variants and the compound heterozygous case, LAT1763, formed a distinct cluster (purple group) due to a consistent phenotype: heterotaxy, right atrial isomerism/asplenia type. This group exhibited atrioventricular connection abnormalities, D-MGA, and pulmonary stenosis/atresia. A grid visualization of HPO annotated proband phenotypes (Additional file 3: Fig.S1A) highlights the severity of the phenotypic spectrum in this cluster. Heterozygous alleles displayed greater phenotypic variability across clusters (Fig. 5), with no unaffected cases reported for biallelic predicted deleterious *NODAL* variants, suggesting potential complete penetrance. Conversely, seven families with heterozygous G260R showed reduced penetrance (Fig. 1A, Additional file 2: Table S7).

The red and olive clusters shared higher phenotypic similarity, primarily featuring DTGA (90%) and DORV (70%) without pulmonary artery/valve atresia. The red cluster differentiated by a prevalence of straddling atrioventricular valve and hypoplastic aortic arch with coarctation of the aorta (57.1%). Notably, 66.7% of probands in the olive cluster exhibited a heterotaxy phenotype. The green and teal clusters were closely related phenotypically, with LTGA (92.3%), L-looping of the right ventricle with discordant atrioventricular connection (84.6%), and CCTGA (76.9%) as predominant features. The green cluster further stood out with pulmonary artery/valve atresia (100%) and dextrocardia as a majority feature (66.7%) (Fig. 5).

### Heterozygous CNV deletions spanning *NODAL* in laterality defects

**Fig. 6:**
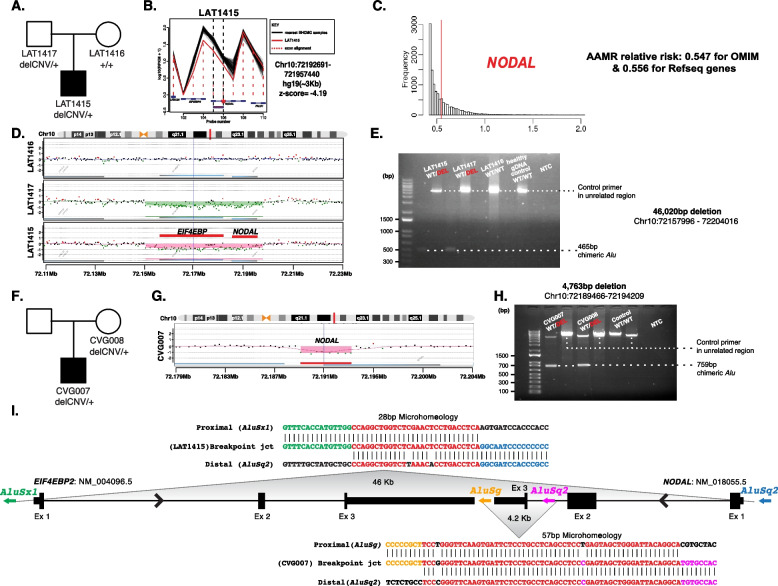
*NODAL* heterozygous copy number variant (CNV) deletion alleles in laterality defect cases. **A** Proband LAT1415 pedigree (Family 30) (delCNV/+ heterozygous, +/+ WT homozygous). **B** HMZDelFinder analyses detected a CNV deletion at the 5′ start of *NODAL* using ES read count data (RPKM); red vertical dashed lines align to each gene exon, horizontal jagged lines (black: controls; red: deletion CNV subject) show distribution of individual read depth values for that given exon. **C** The *Alu-Alu* mediated rearrangement (AAMR) risk score for *NODAL* and *EIF4EBP2* using AluAluCNVpredictor tool. **D** Family 30 aCGH showed a 46-kb deletion in proband and father. **E** The 1% agarose gel electrophoresis of PCR products showing the recombinant junction. The junction primer pair was designed to produce an amplicon size of 465 base pairs for deleted alleles resulting from AAMR with the formation of a chimeric *Alu*. **F** Family 31 pedigree of proband (CVG007). **G** Confirmation by aCGH showed a ~4 kb deletion CNV spanning *NODAL* exon 3. **H** The gel electrophoresis of PCR products showing the recombinant junction with lighter 700 base pair bands representing heterozygous deleted alleles and more intense bands (~4 kb) representing WT allele. **I** A schematic representation of *NODAL* and *EIF4EBP2*. Note convergent transcripts for *NODAL* and *EIF4EBP2* (black arrow heads representing gene’s orientation). Breakpoint sequences for *NODAL* deletions are also shown. The proximal reference sequence and its matching proband breakpoint sequences are shown in green for LAT1415 and orange for CVG007, the distal reference sequence and its matching proband breakpoint sequences are in blue for LAT1415 and purple for CVG007, and microhomology at the junction is shown in red. The 46 kb deletion in LAT1415 presumably results from AAMR between *Alu*Sx1/*Alu*Sq2, whereas the 4 kb deletion in CVG007 proposed to result from AAMR between *Alu*Sg/*Alu*Sq2

Structural genomic variation spanning *NODAL* was assessed in this laterality defect cohort using XHMM and HMZDelFinder CNV detection tools by comparison of ES read depth data [17, 18]. A high-confidence heterozygous deletion was observed in LAT1415 (Fig. 6A, B), who presented with tricuspid atresia, straddling mitral valve (MV), DORV, D-MGA, and severe coarctation of the aorta. ddPCR confirmed paternal inheritance, with the father having no CHD history (Additional file 3: Fig.S3).

The *NODAL* deletion CNV, spanning the entire gene (46 Kb), was further characterized using high-density aCGH, revealing a high instability score (0.547 for OMIM genes, 0.556 for RefSeq genes) through AluAluCNVpredictor [25], suggesting susceptibility to *Alu-Alu*-mediated rearrangement (AAMR; Fig. 6C). Breakpoint junction analysis (Fig. 6E) indicated direct-oriented *AluSx1/AluSq2* elements, consistent with the predicted AAMR-generated event. This deletion likely resulted from a new mutation in a preceding generation within the family (Fig. 6I).

Additionally, a maternally inherited single-exon deletion of *NODAL* was identified in a male patient (CVG0007) with DORV, D-MGA, subaortic stenosis, and severe arch hypoplasia/coarctation (Fig. 6F–H). His mother had no CHD. This patient was recruited through BG, in which the deletion was initially predicted by CMA testing. Breakpoint junction analysis revealed an AAMR event involving directly oriented *AluSg/AluSq2* (Fig. 6I).

## Discussion

NODAL is a key signaling molecule that plays a pivotal role during embryonic patterning, axis formation, and germ layer specification during early developmental stages. NODAL signaling is also known to be involved in the maintenance of human embryonic stem cell pluripotency and differentiation into specific cell types in a context-dependent manner, and regulation of L-R lineage determination [26, 27]. Several animal-model studies including mouse and zebrafish, as well as misexpression studies in chick and Xenopus, have demonstrated the influence of NODAL signaling in heart development along with L-R axis determination [28, 29]. To further our understanding of *NODAL* variants and associated phenotypes, here we investigated *NODAL* variants in 31 families with CHD (Fig. 1). Overall, *NODAL* variants were observed in 6.5% of the laterality defects cohort (21/321), and in 9% (10/111) of Hispanic patients in the cohort, and in 15.6% (10/64) of Hispanic patients in that cohort with *NODAL*-associated laterality defects; signifying the contribution of *NODAL* variation to CHD. Of note, probands in 4 families had biallelic *NODAL* variant alleles (Fig. 1B) and one family had one laterality CHD proband (LAT016) with a de novo *NODAL* SNV and a sibling with CHD associated with a de novo del22q11.2 CNV (Family 18, Fig. 1A). The latter family speaks to both the rates of structural variant mutagenesis in sporadic birth defects and the need to investigate all CHD probands by genomic studies [30].

Although there was a spectrum of CHD lesions associated with *NODAL* variants, three unique patterns were clear. First, all CHD lesions included MGA of some type, whether it be D-transposed or malposed, L-transposed or malposed, or with an anterior aorta and pulmonary atresia in which the exact malposed relationship could not be discerned. This is a critical finding; in that it suggests that CHD lesions with great artery malposition are within the “laterality defect” classification. Historically DTGA, DORV with malposed great arteries, and sometimes CCTGA are often instead classified as conotruncal defects. Despite this, those three lesions are virtually never seen in 22q11.2 deletion syndrome, which is by far the most common cause of conotruncal defects. The lesions tricuspid atresia and DILV have poorly understood genetic etiologies, but multiple cases of both lesions, when present with malposed great arteries, were seen in the cohort. Perhaps separating those with malposed great arteries and those with normal great arteries will help further genetic understanding. Understanding these lesions’ more optimal classification as laterality defects may assist practitioners in pursuing indicated genetic testing, will help variant prioritization and clinical interpretation, and will better guide familial genetic counseling.

Second, almost half of the cohort had left ventricular looping, which is rare even in those with CHD (~2% of CHD [31]), and 14.1% of the study cohort with left ventricular looping had a *NODAL* variant. This is logical given NODAL’s critical role in L-R axis patterning. However, in a clinical setting this strong association has not been well appreciated and is even stronger that the most commonly known association between 22q11.2 deletion syndrome and simple tetralogy of Fallot (in which ~8% have 22q11.2 deletion).

Third, *NODAL* variants when associated with heterotaxy were exclusively associated with either asplenia syndrome/right atrial isomerism (*n* = 10) or in two cases with visceral/bronchial/atrial situs inversus with CHD. Right atrial isomerism and left atrial isomerism are often grouped together as similar lesions and even share the same Van Praagh nomenclature of “atrial situs ambiguous”. However, the distinct lack of left atrial isomerism cases in this *NODAL* cohort and in published literature suggest that genetically and etiologically, these two conditions are distinct and should be evaluated differently when studying genetic mechanisms and inheritance.

The prevalence of *NODAL* variants we observed is relatively high compared to that reported from the Pediatric Cardiac Genomics Consortium (PCGC) CHD cohort where *NODAL* variants were identified in only four cases of 2871 CHD probands (Additional file 2: Tables S9 and S10) [32]. However, this report was limited to de novo and recessive variant alleles, did not include copy number variants, had a heterogeneous collection of CHD, had a reporting threshold too restrictive to report the G260R variant, and only included 280 patients of Hispanic ancestry (9.8% of the reported). If one limits the study to the 523 laterality CHD cases, *NODAL* variants account for 0.76% of cases. In our 321-person laterality CHD cohort with 35% Hispanic ethnicity, if the G260R variant and copy number variants are not included, the yield would be much less, with only 10 cases with a *NODAL* variant (3.1%). Of note, the phenotypes within the PCGC cohort are completely consistent with our observations, with one simple DTGA, one DORV with D-MGA, and two cases of CCTGA (Additional file 2: Table S10).

The identified *NODAL* variants in our cohort include eleven SNVs (8 missense, 2 splice site, and 1 nonsense), one recurrent complex indel variant (p.R234_P241delinsLTS), and two CNV deletion alleles (one whole *NODAL* gene deletion and one exon 3 deletion). Among the identified SNVs, two (p.M1T, p.L347F) were previously unreported variant alleles that have never been associated with laterality defects and are strongly predicted as disease causing using in silico tools.

The complex indel variant c.700_723delins, p.R234_P241delinsLTS, consisting of a 24-base deletion with a 9 base insertion affects the likely cleavage site of the NODAL protein, affecting its recognition by proprotein convertases and impairing protein maturation. While the exact furin cleavage site in NODAL has not been described to our knowledge, the Arginine-Histidine-Arginine-Arginine (RHRR) sequence, specifically between amino acids 234 and 237 in the precursor form of the protein, represents the only amino acid sequence in NODAL that matches the RXXR motif furin cleaves (Additional file 3: Fig. S4). Secondary structure predictions suggest that the variant may also alter the RNA molecule’s folding and stability, potentially impacting gene expression and developmental processes. These structural changes may affect the accessibility of the RNA molecule to other molecules involved in its processing and function. Overall, the complex allele is likely to have significant effects on RNA and protein structure, as well as gene function, contributing to the observed phenotypic abnormalities associated with laterality defects. However, expression data on cell lines expressing this mutation would be required to confirm changes in gene expression.

In the current cohort, the G260R variant was found in a high prevalence. Four probands with biallelic variation, three homozygotes and one compound heterozygote, were identified. The population specificity of the G260R variant to Hispanics may be due to founder effects resulting from genetic drift. Although specific country of origin ancestry is not available for most patients in this study (only available for 4 of the Hispanic patients which were Mexican), the largest proportion of Hispanics in Texas are of Mexican heritage. None of the probands were known to be related. While formal testing for interrelatedness was not performed, detailed three plus generation family histories were collected both for the study and in the clinical setting and did not suggest any overlap. Additionally, there were no common surnames across the families. Moreover, using an in house tool analyzing the exome data we checked the absence of heterozygosity (AOH)/runs of homozygosity (ROH) regions, especially around this locus, for all the probands with *NODAL* variants in our study. The AOH/ROH data flanking the G260R locus further supports the low potential of consanguinity in our cohort, given that no AOH regions were observed in this region. However, the detection of this variant in unaffected parents suggests that the variant may not always lead to the development of the condition when in heterozygous state, indicating incomplete penetrance. These findings are consistent with the low recurrence risk and complex inheritance pattern observed in most sporadic cases of laterality defects, which suggests that multiple genetic and environmental factors may contribute to the development of the condition. In other words, the level of NODAL function, due to both genetic and environmental perturbations, may govern penetrance and phenotypic severity of CHD phenotypes. The complete penetrance and consistency of phenotype observed for probands with biallelic predicted deleterious variation in *NODAL* supports this hypothesis.

One genetic factor that may affect the penetrance of the G260R allele in certain populations is modifying background genetic variation, which is supported by the observation of higher heterotaxy incidence in the African American and Hispanic populations [33]. The same study also pointed to another factor affecting observed heterotaxy rates, namely diabetes [33], which may impact penetrance of heterotaxy phenotypes through physiologic factors such as exposure of the developing fetus to insulin [34]. Of note, penetrance can be influenced by ancestry-specific haplotypes in congenital scoliosis associated with developmental hemivertebrae defects of the spine [35].

G260R was originally described in 2009 by Mohapatra et al. as pathogenic and causative for heterotaxy with reduced penetrance, variable expressivity, and predominantly affecting Hispanic individuals. Since then, G260R has been found in several individuals with CHD and/or heterotaxy across multiple centers (https://www.ncbi.nlm.nih.gov/clinvar/variation/8269/). In ClinVar, G260R has been classified as “conflicting interpretations of pathogenicity” based on the American College of Medical Genetics and Genomics (ACMG) criteria (ClinVar Accession: VCV000008269.9). It is noteworthy that the G260R variant demonstrates a CADD score of 25.5 and a REVEL score of 0.793, both indicative of its potential pathogenic impact. However, the reclassification of the *NODAL* G260R allele to an ACMG classification of LP, as proposed in this study, should be considered within the context of our laterality defects cohort. The high prevalence of this variant in our cohort, along with the presence of biallelic cases within our study, supports this reclassification. Furthermore, the implications of this reclassification should be considered in the broader clinical and genetic counseling context, including the potential for preimplantation genetic testing for LP variants.

Quantitative phenotypic analysis showed high phenotypic similarity score between all clusters suggesting no specific genotypic-phenotypic correlations. However, all biallelic cases with G260R variant were shown to have the highest phenotypic similarity between probands suggesting the most consistent phenotype (Fig. 5 and Additional file 2: Table S6). This finding provides evidence for a genotypic-phenotypic correlation and an allele-specific gene dosage model [36]. However, it is important to note that the absence of such a correlation among individuals without the variant does not necessarily indicate the absence of other genetic factors influencing the phenotype. It is also important to consider other factors that may contribute to the observed phenotype, such as environmental factors and epigenetic modifications.

We report two unrelated laterality defect cases whose underlying disease-causing mutations were *NODAL* CNV deletion alleles: a whole gene deletion and a single exon deletion. The CNV deletion alleles were shown to most likely have formed due to AAMR events, which is consistent with the high relative gene/genomic instability score (0.547 for OMIM genes and 0.556 for RefSeq genes) observed for *NODAL* using the AluAluCNVpredictor [25]. These observations suggest that *NODAL* CNV deletions should be considered in genomic diagnostics, genetic counseling, and testing for laterality defects.

## Conclusions

Collectively, our findings suggest that assessment of all variant types (SNV, indel, and CNV) in heterotaxy cases can increase molecular diagnosis rates in CHD cases and that allele-specific gene dosage can be an important contributor to penetrance and variable expression of CHD. We confirm that rare variants in *NODAL* contribute to the development of heterotaxy spectrum congenital heart defects [13] and provide evidence that the population specificity of these variants should be taken into consideration in genetic counseling and clinical genomic testing for this condition. Moreover, our study uncovers unreported *NODAL* mutations and mutation types in association with laterality defects, enabling an allelic series that furthers our understanding of the biological perturbations and genetic pathobiology underlying laterality defects. These findings have important implications for the diagnosis and treatment of human laterality defects.

### Supplementary Information

**Additional file 1: Table S1.** Molecular, Cohort, and Phenotypic information on all cases with NODAL variants. **Table S2.** Inclusion criteria and description of included patient groups. **Table S3.** Detailed clinical information for all 33 CHD cases in the study. **Table S4.** CHD gene list analyzed in our cohort. **Table S5.** HPO terms used for all CHD cases in the study.

**Additional file 2: Table S6.** DdPCR primer design for the *NODAL* gene and *RPP30* control gene. **Table S7.** Phenotype for of all cases with p.G260R variation including heterozygous and homozygous cases. **Table S8.** Allele Frequency comparison of patients with G260R NODAL variant. **Table S9.** NODAL variation in PCGC cohort. **Table S10.** Summarized Clinical Information of cases in PCGC Cohort.

**Additional file 3: Figure S1.** *NODAL* Phenotype Grid Comparison – (A) A grid of proband phenotypes was generated using HPO annotated term sets for each proband and ordered based off Hierarchical Agglomerative Clustering of proband phenotypic similarity scores. Probands and variants are labeled at left and color coded by clusters. Colors for each cluster match those displayed in the heatmap. HPO terms are displayed at the bottom of the grid. Within the grid, red denotes presence of a phenotype, while grey denotes absence or lack of clinical data of a phenotype. Frequency for each HPO phenotype in the *NODAL* cohort is shown by the distribution bar graph at top. An asterisk at the right end of individual proband sample number identifier denotes probands found to have biallelic variants in *NODAL*. (B) A grid of proband phenotypes was generated using HPO annotated term sets for each proband with atrial septal defect, ventricular septal defect, single ventricle, and secundum atrial septal defect included (bottom) for comparison to the grid of proband phenotypes presented in (A). Colors from the analysis with atrial septal defect, ventricular septal defect, single ventricle, and secundum atrial septal defect removed are preserved to show differences in clustering between the analyses with and without these terms. **Figure S2.** Gap Statistic Curve – Gap statistic results for hierarchical clustering of the distance matrix generated from the similarity matrix of pairwise proband phenotype similarity scores is shown. The gap statistic is shown on the y-axis and the number of clusters considered is shown on the x-axis. The slope of the curve is steepest before 5 clusters, and so 5 was chosen for the number of clusters to group *NODAL* proband phenotypes into. **Figure S3.** Copy number analysis of *NODAL* by Droplet-digital PCR (ddPCR) for families 30 and 31. (A) The deletion of *NODAL* was found in the proband (LAT1415) and father (LAT1417) in this pedigree (family 30). Analysis of the mother (LAT1416) and a healthy unrelated control shows normal copy number. (B) The deletion of *NODAL* exon 3 was found in the proband (CVG007) and mother (CVG008) in this pedigree (family 31). Father’s DNA was not available for testing. **Figure S4.** Diagrams of the NODAL preproprotein predicted structure highlighting the Furin/PACE4 cleavage site motif. (A) Wild type NODAL tertiary structure model generated by https://alphafold.ebi.ac.uk/entry/Q96S42, with a zoomed in view at the Furin/PACE4 cleavage site motif (RHRR) in the context of the protein structure. (B) Simplified illustration of the NODAL primary structure of the wild type (top diagram) showing the presence of the Furin/PACE4 cleavage site motif. The deleted amino acids p.R234_P241del (middle diagram), and the mutated p.R234_P241delinsLTS form (bottom diagram) showing the disrupted Furin/PACE4 cleavage site.

## Data Availability

The datasets used and/or analyzed during the current study are available in the supplementary data.

## Abbreviations

L-R: Left-right
CHD: Congenital heart disease
DORV: Double outlet right ventricle
AVCD: Atrioventricular canal defect
TGA: Transposition of the great arteries
L-LPM: Left-lateral plate mesoderm
R-LPM: Right-lateral plate mesoderm
ES: Exome sequencing
P/LP: Pathogenic/likely pathogenic
CMA: Clinical microarray
BG: Baylor Genetics
TGP: 1000 Genomes Project
ARIC: Atherosclerosis Risk in Communities Study Database
CADD: Combined Annotation Dependent Depletion
LB: Luria-Bertani
HAC: Hierarchical Agglomerative Clustering
ddPCR: Droplet digital PCR
aCGH: Array comparative genomic hybridization
LR-PCR: Long-range PCR
CCTGA: Congenitally corrected transposition of the great arteries
LTGA: Levo-transposition of the great arteries
DTGA: Dextro-transposition of the great arteries
D-MGA: D-malposed great arteries
PS: Pulmonary stenosis
AVMs: Arteriovenous malformations
VUR: Vesicoureteral reflux
WT: Wild-type
MV: Mitral valve
AAMR: *Alu*-*Alu* Mediated rearrangement
PCGC: Pediatric Cardiac Genomics Consortium
ACMG: American College of Medical Genetics and Genomics
