# Supplementary material for: NODAL variants are associated with a continuum of laterality defects from simple D-transposition of the great arteries to heterotaxy
Source: Genome Med. 2024 Apr 3;16:53. doi: 10.1186/s13073-024-01312-9 (PMC10988827; doi:10.1186/s13073-024-01312-9)
Supplement: Supplementary file 2 — Additional file 2: Table S6. DdPCR primer design for the NODAL gene and RPP30 control gene. Table S7. Phenotype for of all cases with p.G260R variation including heterozygous and homozygous cases. Table S8. Allele Frequency comparison of patients with G260R NODAL variant. Table S9. NODAL variation in PCGC cohort. Table S10. Summarized Clinical Information of cases in PCGC Cohort. [file 13073_2024_1312_MOESM2_ESM.docx]

**Table S1-S5:** Additional file 1.

Table S6: DdPCR primer design for the *NODAL* gene and *RPP30* control gene

|  | **Left primer** | **Right primer** | **Tm** |
| --- | --- | --- | --- |
| *NODAL*-LAT1415 | CTGTCCTGGGGCTGTTGAG | TGGCCTGTTACTCTCACCCT | 60 |
| *NODAL* -CVG0007 | ACCCACATTCTTCCACGATCA | GTGCCCCAGTGAAGACCAAG | 60 |
| *RPP30* | CGGATCCATCTCACTGCAAT | CATTTTCCCTCGCCCCCGCCAAACC | 60 |

**Table S7** Phenotype for of all cases with p.G260R variation including heterozygous and homozygous cases.

| **ID** | **Case** | **Family** | **Zyg.** | **DNA change** | **Protein** | **Race/ Ethn** | **Sex** | **Seg. anatomy** | **CHD summary** | **Arrhythmia by last FU** |
| --- | --- | --- | --- | --- | --- | --- | --- | --- | --- | --- |
| Heterozygotes | | | | | | | | | |  |
| LAT0909 | Proband | 19 | Het | c.778G>A | p.G260R | NHW | M | S,D,D | DTGA | None |
| LAT1391 | Proband | 21 | Het | c.778G>A | p.G260R | H | M | S,D,D | DTGA, VSD, CoA | None |
| LAT0858 | Proband | 17 | Het | c.778G>A | p.G260R | H | M | S,D,D | Straddling MV, VSD, DORV with D-MGA, PS | AT |
| LAT1016 | Proband | 18 | Het | c.778G>A | p.G260R | H | M | S,D,D | Straddling MV, VSD, DORV with D-MGA, CoA | None |
| LAT1028 | Proband | 20 | Het | c.778G>A | p.G260R | H | F | S,D,L | DILV, VSD, Infundibular PA, Aorta left of MPA | None |
| LAT0201 | Proband | 13 | Het | c.778G>A | p.G260R | H | F | S,L,X | L ventricular looping, DILV, Infundibular PA, Aorta right of MPA | IART |
| LAT1617 | Proband | 22 | Het | c.778G>A | p.G260R | H | M | S,L,L | L ventricular looping, VSD, PA, Aorta left of MPA | AT |
| LAT1769 | Proband | 23 | Het | c.778G>A | p.G260R | H | M | S,L,L | L ventricular looping, LTGA, Pulmonary stenosis | CAVB w/PM |
| CVG0001 | Proband | 11 | Het | c.778G>A | p.G260R | H | M | S,L,L | L ventricular looping, MV atresia, VSD, Infundibular PA, Aorta left of MPA | AT |
| LAT0830 | Proband | 16 | Het | c.778G>A | p.G260R | H | F | S,L,D | L ventricular looping, Straddling MV, VSD, DORV with D-MGA, subAS, CoA | None |
| LAT0040 | Proband | 12 | Het | c.778G.A | p.G260R | H | F | I,D,D | Situs Inversus, VSD, PA, discontinuous pulmonary arteries | AT |
| LAT0248 | Proband | 14 | Het | c.778G>A | p.G260R | H | M | A,D,D | Heterotaxy (asplenia), RV-dominant CAVC, DORV with D-malposed GA | WPW w/ SVT, Sinus node dysfunction w/PM |
| LAT0658 | Proband | 15 | Het | c.778G>A | p.G260R | H | M | A,?,X | Heterotaxy (asplenia), Infundibular PA | None |
| LAT1764 | Mother | 7 | Het | c.778G>A | p.G260R | H | F |  | None known (no exam) | None known |
| CVG0010 | Mother | 8 | Het | c.778G>A | p.G260R | H | F |  | None known (no exam) | None known |
| CVG0009 | Father | 8 | Het | c.778G>A | p.G260R | H | M |  | None known (no exam) | None known |
| LAT0109 | Mother | 9 | Het | c.778G>A | p.G260R | H | F |  | None known (no exam) | None known |
| LAT0110 | Father | 9 | Het | c.778G>A | p.G260R | H | M |  | None known (no exam) | None known |
| LAT0266 | Mother | 10 | Het | c.778G>A | p.G260R | H | F |  | None known (no exam) | None known |
| LAT0267 | Father | 10 | Het | c.778G>A | p.G260R | H | M |  | None known (no exam) | None known |
| LAT0043 | Mother | 12 | Het | c.778G>A | p.G260R | H | F |  | None known (no exam) | None known |
| LAT0202 | Mother | 13 | Het | c.778G>A | p.G260R | H | F |  | None known (no exam) | None known |
| LAT0250 | Father | 14 | Het | c.778G>A | p.G260R | H | F |  | None known (no exam) | None known |
| LAT0832 | Father | 16 | Het | c.778G>A | p.G260R | H | M |  | None known (no exam) | None known |
| LAT1030 | Father | 20 | Het | c.778G>A | p.G260R | H | M |  | None known (no exam) | None known |
| LAT1393 | Father | 21 | Het | c.778G>A | p.G260R | H | M |  | None known (no exam) | None known |
| LAT1619 | Father | 22 | Het | c.778G>A | p.G260R | H | M |  | None known (no exam) | None known |
| Homozygotes | | | | | | | | | |  |
| LAT1763 | Proband | 7 | Comp Het | c.G778A  c.700_723delinsTCGACTTCC | p.G260R  - | H | F | A,D,D | Heterotaxy (asplenia), DILV, VSD, Infundibular PA, Aorta right of MPA | None |
| CVG0006 | Proband | 8 | Homo | c.778G>A | p.G260R | H | M | A,D,D | Heterotaxy (asplenia), CAVC, Infundibular PA, Aorta right of MPA | None |
| LAT0108 | Proband | 9 | Homo | c.778G>A | p.G260R | H | M | A,D,D | Heterotaxy (asplenia), RV-dominant CAVC, Infundibular PA, MAPCAs | None |
| LAT0265 | Proband | 10 | Homo | c.778G>A | p.G260R | H | M | A,?,D | Heterotaxy (asplenia), CAVC DORV with D-MGA, PS | AT |

CAVB: Complete atrioventricular block; CAVC: Common atrioventricular septal defect; CNV: Copy number variant; CoA: Coarctation; DILV: Double inlet left ventricle; D-MGA: Dextro-malposed great artries; DORV: Double outlet right ventricle; DTGA: Dextro-transposition of the great arteries; Ethn.: Ethnicity; F: Female; GA: Great arteries; H: Hispanic; Het.: Heterozygous; Homo.: Homozygous; IART: Intra-atrial re-entrant tachycardia; LTGA: Left transposition of the great arteries; IART: Intra-atrial reentry tachycardia; L-ventricular: left ventricular; M: Male; MAPCAs: Multiple aorto-pulmonary collateral arteries; MPA: Main pulmonary artery; MV: mitral valve; NHA: Non-Hispanic Asian; NHW: Non-Hispanic White; PA: Pulmonary atresia; PM: pacemaker; PS: Pulmonary stenosis; RV: right ventricular; Seg: Segmental; SubAS: Subaortic stenosis; TV: Tricuspid valve; VSD: Ventricular septal defect; WPW: Wolfe-Parkinson-White; Zyg.: Zygosity

|  | Group 1a (Laterality CHD probands) | | | | | | | gnomAD | | | |  | |
| --- | --- | --- | --- | --- | --- | --- | --- | --- | --- | --- | --- | --- | --- |
|  | G260R patients | Total patients | % of patients | G260R allele count | Allele number | Homo. patients | Allele frequency | G260R allele count | Allele number | Homo. patients | Allele frequency | Odds Ratio (95%CI) | p |
| All | 11 | 321 | 3.4% | 13 | 642 | 2 | 0.0202 | 78 | 251,459 | 0 | 0.00031 | 66.6 (36.8-120.4) | <0.0001 |
| Hispanic/Latino | 10 | 111 | 9.0% | 12 | 222 | 0 | 0.0541 | 76 | 34,592 | 0 | 0.00219 | 26.0 (13.9-48.4) | <0.0001 |
| Non-Hispanic White/ European (Non-Finnish) | 1 | 157 | 0.6% | 1 | 314 | 0 | 0.0032 | 1 | 113,740 | 0 | 0.00000879 | 3463.4 (22.7-5822.8) | <0.0001 |
| Non-Hispanic Black/African | 0 | 24 | 0.0% | 0 | 48 | 0 | 0 | 0 | 16,254 | 0 | 0 | - | - |
| Non-Hispanic Asian | 0 | 12 | 0.0% | 0 | 24 | 0 | 0 | 0 | 49,010 | 0 | 0 | - | - |
| **By Lesion** | | | | | | | |  |  |  |  |  |  |
| Simple DTGA | 2 | 49 | 4.1% | 2 | 98 | 0 | 0.0204 |  |  |  |  |  |  |
| DORV with malposed GA | 0 | 26 | 0.0% | 0 | 52 | 0 | 0.0000 |  |  |  |  |  |  |
| CCTGA | 2 | 26 | 7.7% | 2 | 52 | 0 | 0.0384 |  |  |  |  |  |  |
| DILV, All | 2 | 33 | 6.1% | 2 | 66 | 0 | 0.0303 |  |  |  |  |  |  |
| DILV, D-looped | 1 | 11 | 9.1% | 1 | 22 | 0 | 0.0454 |  |  |  |  |  |  |
| DILV, L-looped | 1 | 22 | 4.5% | 1 | 44 | 0 | 0.0227 |  |  |  |  |  |  |
| Any L-looping | 4 | 66 | 6.1% | 4 | 132 | 0 | 0.0303 |  |  |  |  |  |  |
| Heterotaxy, Situs Inversus w/ CHD | 0 | 15 | 0.0% | 0 | 30 | 0 | 0.0000 |  |  |  |  |  |  |
| Heterotaxy, Right atrial isomerism/ Asplenia syndrome | 4 | 68 | 5.9% | 6 | 136 | 2 | 0.0441 |  |  |  |  |  |  |
| **By Lesion, Limited to Hispanic** | | | | | | | |  |  |  |  |  |  |
| Simple DTGA | 1 | 19 | 5.6% | 1 | 38 | 0 | 0.0263 |  |  |  |  |  |  |
| DORV with malposed GA | 0 | 10 | 0.0% | 0 | 20 | 0 | 0.000 |  |  |  |  |  |  |
| CCTGA | 2 | 8 | 25.0% | 2 | 16 | 0 | 0.125 |  |  |  |  |  |  |
| DILV, All | 2 | 11 | 18.2% | 2 | 22 | 0 | 0.0909 |  |  |  |  |  |  |
| DILV, D-looped | 1 | 2 | 50.0% | 1 | 4 | 0 | 0.250 |  |  |  |  |  |  |
| DILV, L-looped | 1 | 9 | 11.1% | 1 | 18 | 0 | 0.0555 |  |  |  |  |  |  |
| Any L-looping | 4 | 23 | 17.4% | 4 | 46 | 0 | 0.0870 |  |  |  |  |  |  |
| Heterotaxy, Situs Inversus w/ CHD | 0 | 4 | 0.0% | 0 | 6 | 0 | 0.000 |  |  |  |  |  |  |
| Heterotaxy, Right atrial isomerism/ Asplenia syndrome | 4 | 26 | 27.3% | 6 | 52 | 2 | 0.1154 |  |  |  |  |  |  |

**Table S8: Allele Frequency comparison of patients with G260R *NODAL* variant**

Homo.: Homozygous

**Table S9: *NODAL* variation in PCGC Cohort**

| **Identifier** | **Family** | **Report*** | **Sex** | **Ethn.** | **Inh.** | **Zyg.** | **Genomic Position** | **C change** | **P change** | **Mut.** | **gnomAD** | **CADD_**  **phred** | **REVEL** | **ClinVar** |
| --- | --- | --- | --- | --- | --- | --- | --- | --- | --- | --- | --- | --- | --- | --- |
| 1-02769 | Family 1 | PCGC | Unk | Unk | Unk | Het | 10:72195041; C>T | c.891+1G>A | - | sp | . | 33 | - | LP |
| 1-00034 | Family 2 | PCGC | Unk | Unk | Unk | Het | 10:72201382; C>T | c.42G>A | p.W14X | ns | . | . |  | NF |
| 1-01626 | Family 3 | PCGC | Unk | Unk | Unk | Het | 10:72195547; A>T | c.386T>A | p.L129X | ns | . | . |  | NF |
| 1-10098 | Family 4 | PCGC | Unk | Unk | Unk | Het | 10:72195098; C>A | c.835G>T | p.E279X | ns | . | . |  | NF |
| * Jin *et al.* (2017) Nat Genet.  Ethn., ethnicity; Het, heterozygous; Inh., inheritance; LP, Likely Pathogenic; NF, not found; ns, nonsense; Mut., mutation type; sp, splicing; Unk, unknown; Zyg., zygosity | | | | | | | | | | | | | | |

**Table S10: Summarized Clinical Information of cases in PCGC Cohort**

|  |  |  | **D-looped ventricles** | | | **L-looped ventricles** | | | **Systemic laterality condition** | | **No CHD** |
| --- | --- | --- | --- | --- | --- | --- | --- | --- | --- | --- | --- |
| **Sample ID** | **Report*** | **Seg. anatomy** | **Simple DTGA** | **DORV with D- malposed GA** | **Tricuspid atresia with malposed GA** | **CCTGA** | **DILV** | **Other left ventricular looping lesion** | **Heterotaxy (right atrial isomerism/ asplenia)** | **Situs inversus** | **No CHD** |
| 1-02769 | PCGC | S,L,L |  |  |  |  |  | X |  |  |  |
| 1-00034 | PCGC | S,D,D |  | X |  |  |  |  |  |  |  |
| 1-01626 | PCGC | S,L,L |  |  |  |  |  | X |  |  |  |
| 1-10098 | PCGC | S,D,D | X |  |  |  |  |  |  |  |  |
| *Jin *et al.* (2017) Nat Genet.  DILV: Double inlet left ventricle; D-malposed: Dextro-malposed; DORV: Double outlet right ventricle; DTGA: Dextro-transposition of the great arteries; GA: Great arteries; Het.: Heterozygous; L-ventricular: left ventricular; S: Situs Solitus; Seg: Segmental; Unk: unknown; Zyg.: Zygosity. | | | | | | | | | | | |
