# Supplementary figures and images for: NODAL variants are associated with a continuum of laterality defects from simple D-transposition of the great arteries to heterotaxy
Source: Genome Med. 2024 Apr 3;16:53. doi: 10.1186/s13073-024-01312-9 (PMC10988827; doi:10.1186/s13073-024-01312-9)

Fig S1

A

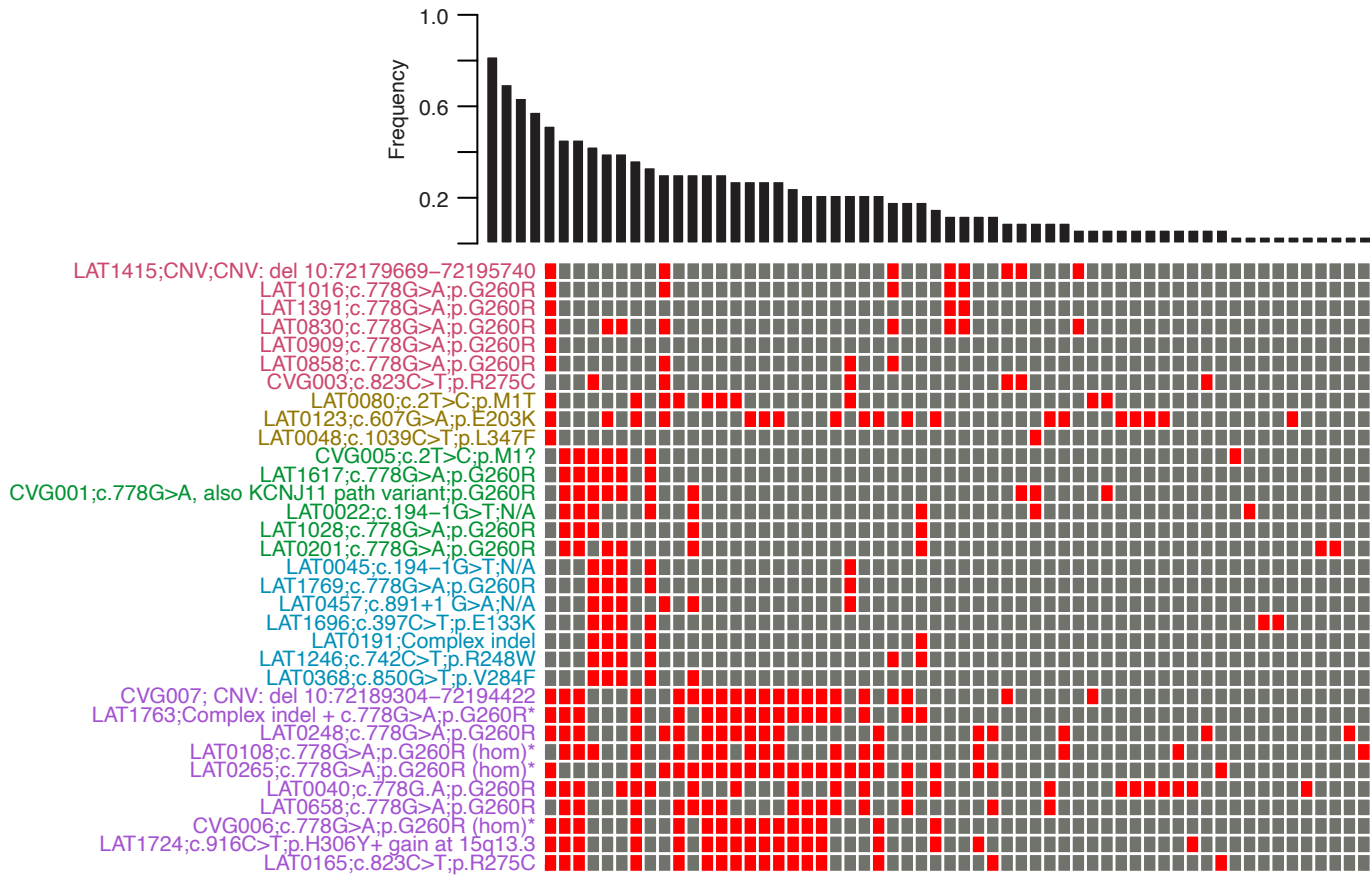

B

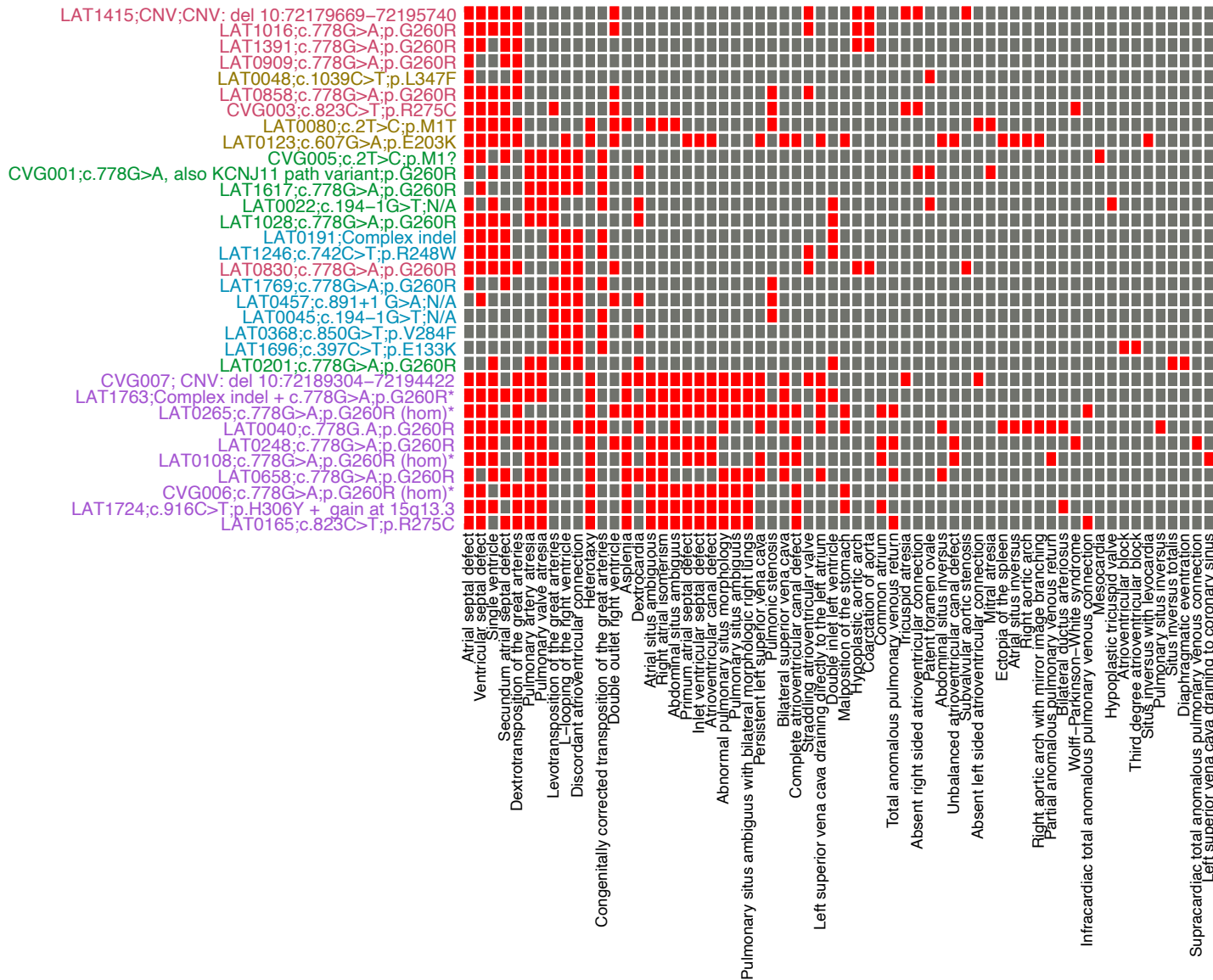

Fig S2

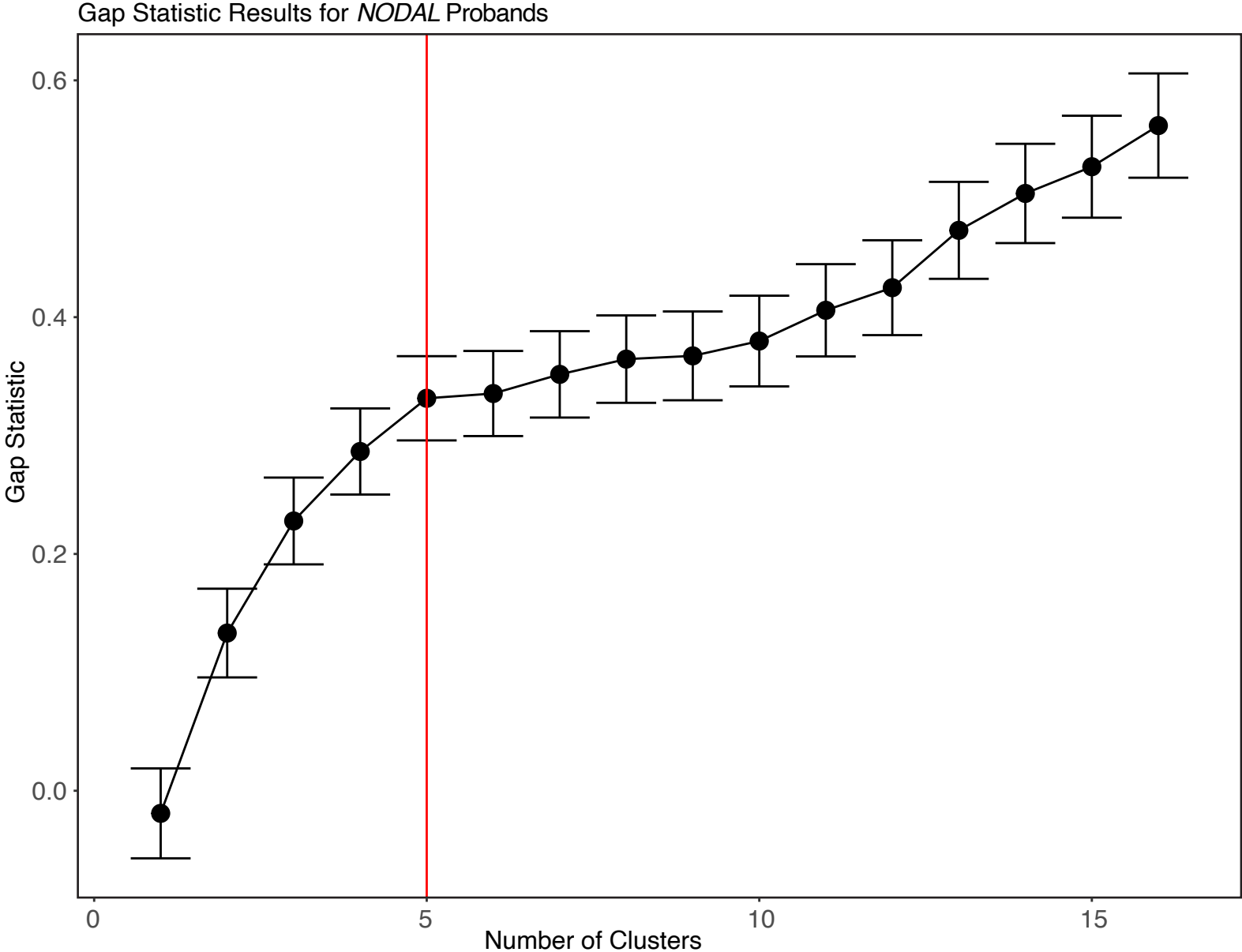

Fig S3

A.

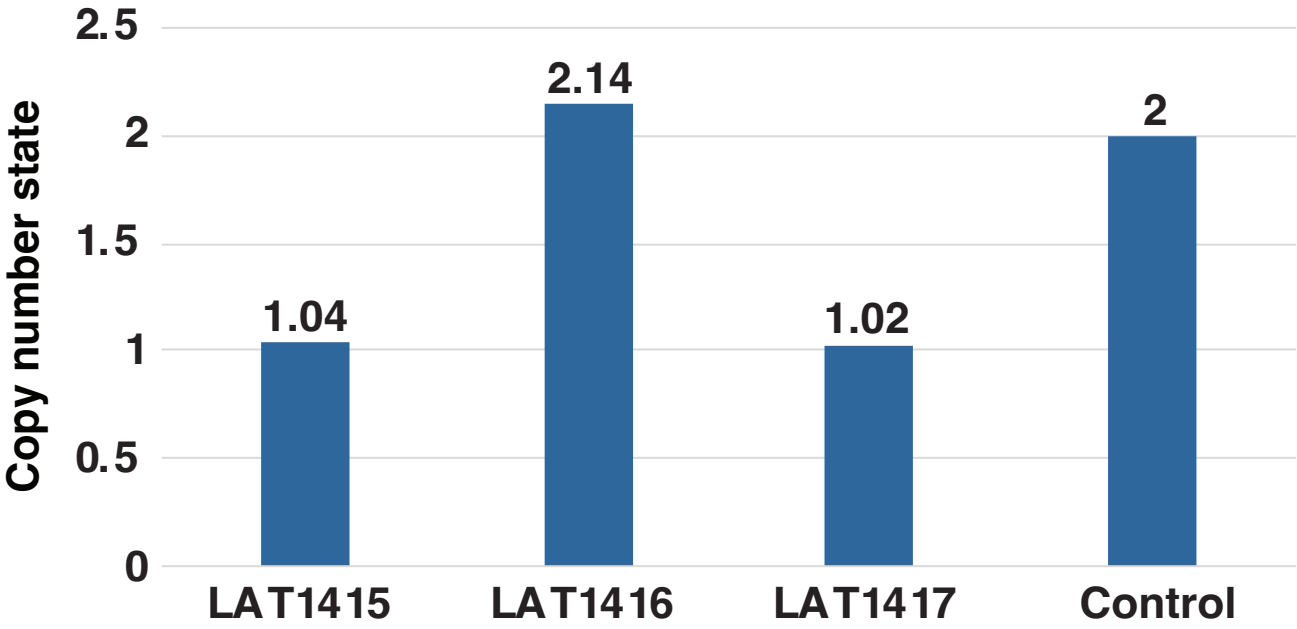

B.

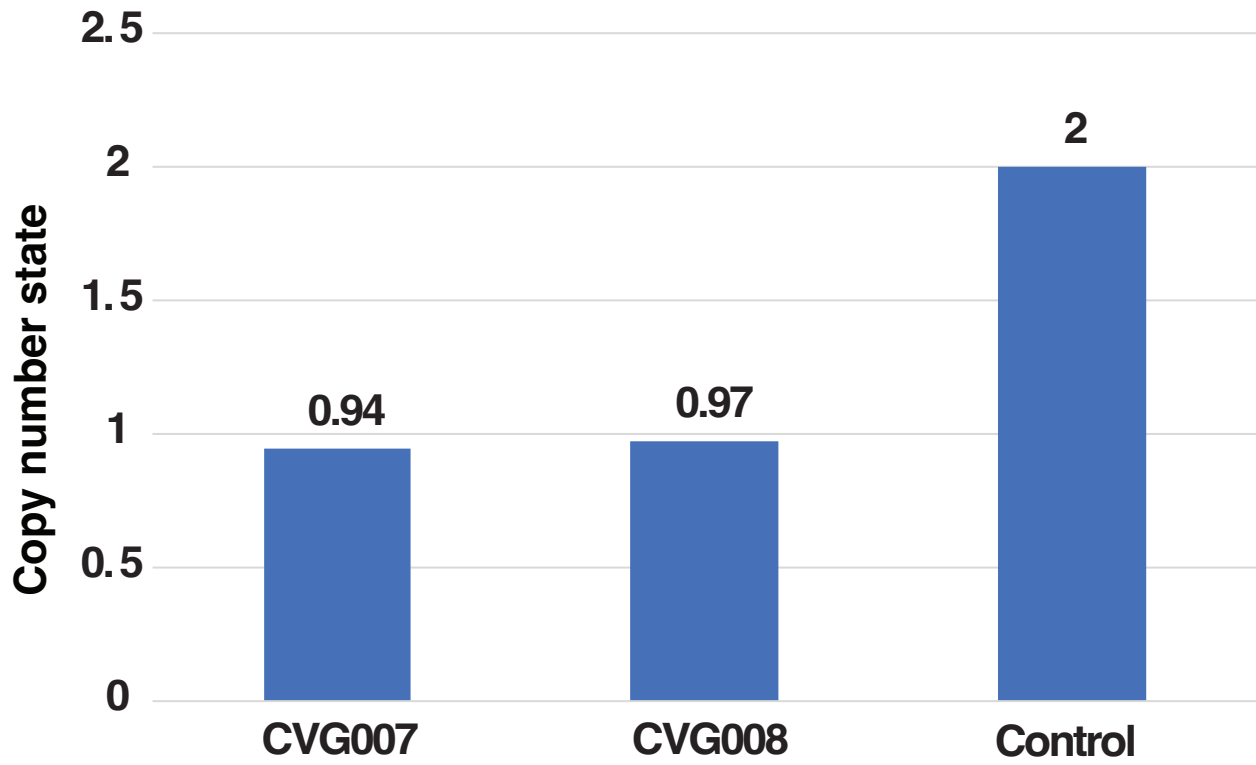

**A**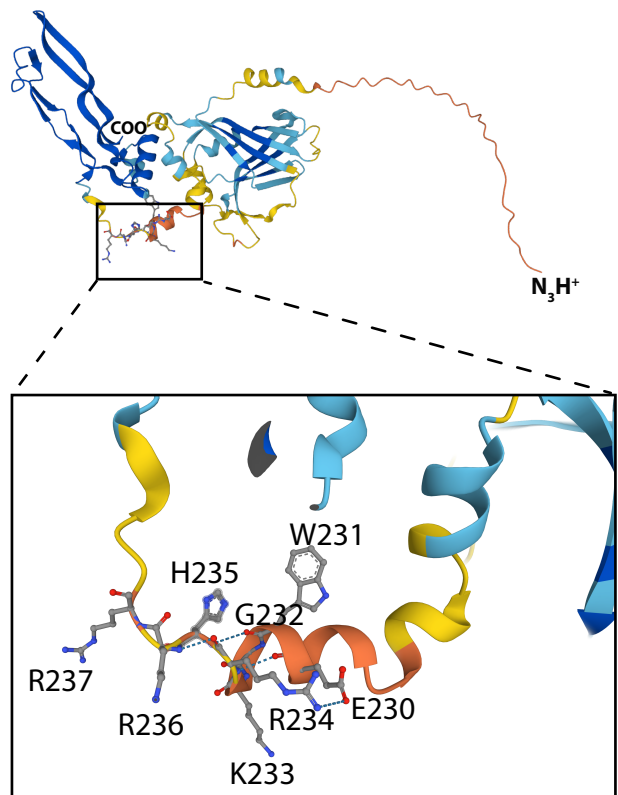**B**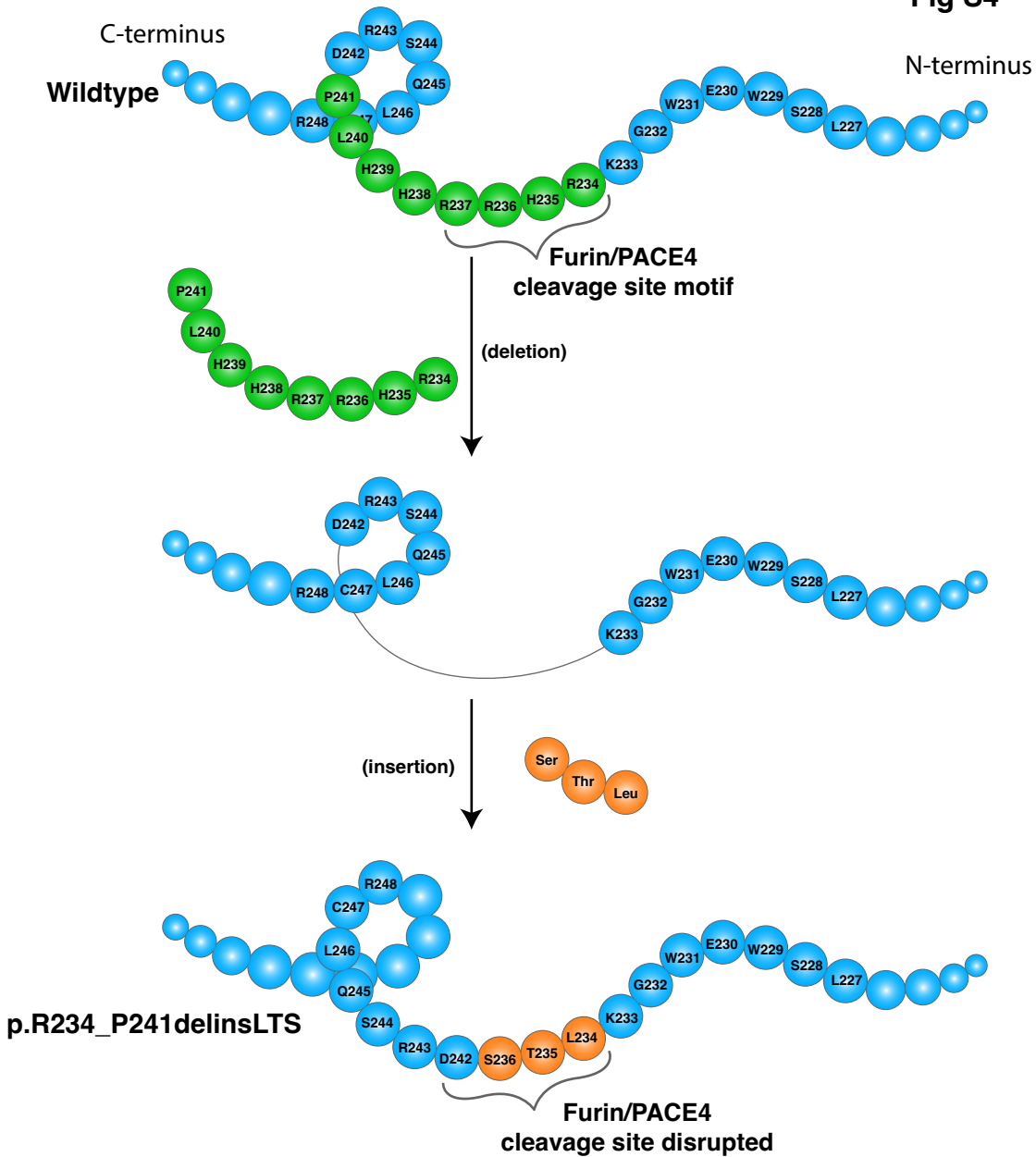

Supplement: Supplementary file 3 — Additional file 3: Figure S1. NODAL Phenotype Grid Comparison – (A) A grid of proband phenotypes was generated using HPO annotated term sets for each proband and ordered based off Hierarchical Agglomerative Clustering of proband phenotypic similarity scores. Probands and variants are labeled at left and color coded by clusters. Colors for each cluster match those displayed in the heatmap. HPO terms are displayed at the bottom of the grid. Within the grid, red denotes presence of a phenotype, while grey denotes absence or lack of clinical data of a phenotype. Frequency for each HPO phenotype in the NODAL cohort is shown by the distribution bar graph at top. An asterisk at the right end of individual proband sample number identifier denotes probands found to have biallelic variants in NODAL. (B) A grid of proband phenotypes was generated using HPO annotated term sets for each proband with atrial septal defect, ventricular septal defect, single ventricle, and secundum atrial septal defect included (bottom) for comparison to the grid of proband phenotypes presented in (A). Colors from the analysis with atrial septal defect, ventricular septal defect, single ventricle, and secundum atrial septal defect removed are preserved to show differences in clustering between the analyses with and without these terms. Figure S2. Gap Statistic Curve – Gap statistic results for hierarchical clustering of the distance matrix generated from the similarity matrix of pairwise proband phenotype similarity scores is shown. The gap statistic is shown on the y-axis and the number of clusters considered is shown on the x-axis. The slope of the curve is steepest before 5 clusters, and so 5 was chosen for the number of clusters to group NODAL proband phenotypes into. Figure S3. Copy number analysis of NODAL by Droplet-digital PCR (ddPCR) for families 30 and 31. (A) The deletion of NODAL was found in the proband (LAT1415) and father (LAT1417) in this pedigree (family 30). Analysis of the mother [file 13073_2024_1312_MOESM3_ESM.pdf]
